# A chromosome-level genome assembly of an alpine plant *Crucihimalaya lasiocarpa* provides insights into high-altitude adaptation

**DOI:** 10.1093/dnares/dsac004

**Published:** 2022-01-29

**Authors:** Landi Feng, Hao Lin, Minghui Kang, Yumeng Ren, Xi Yu, Zhanpeng Xu, Shuo Wang, Ting Li, Wenjie Yang, Quanjun Hu

**Affiliations:** Key Laboratory of Bio-Resource and Eco-Environment of Ministry of Education, College of Life Sciences, Sichuan University, Chengdu 610065, China

**Keywords:** *Crucihimalaya*, adaptation, *de novo* genome, karyotype evolution, high altitude

## Abstract

It remains largely unknown how plants adapt to high-altitude habitats. *Crucihimalaya* (Brassicaceae) is an alpine genus occurring in the Qinghai–Tibet Plateau characterized by cold temperatures and strong ultraviolet radiation. Here, we generated a chromosome-level genome for *C. lasiocarpa* with a total size of 255.8 Mb and a scaffold N50 size of 31.9 Mb. We first examined the karyotype origin of this species and found that the karyotype of five chromosomes resembled the ancestral karyotype of the Brassicaceae family, while the other three showed strong chromosomal structural variations. In combination with the rough genome sequence of another congener (*C. himalaica*), we found that the significantly expanded gene families and positively selected genes involved in alpine adaptation have occurred since the origin of this genus. Our new findings provide valuable information for the chromosomal karyotype evolution of Brassicaceae and investigations of high-altitude environment adaptation of the genus.

## 1. Introduction

Alpine plants occurring in high-altitude habitats with cold temperatures and strong ultraviolet (UV) radiation have evolved special adaptations to withstand these abiotic stresses.^1–3^ Genomic studies of non-model alpine species have provided great insights into the genetic mechanisms underlying such adaptations. For example, genomic comparisons of two *Eutrema* (Brassiaceae) species distributed in the high or the low altitude^4^ suggest that gene family expansions and duplicated genes in the alpine species are involved in their disease resistance, DNA damage repair, reproduction and cold tolerance. Further population genomic analyses of the other species with contrasted altitudinal distributions also identified the positively selected genes related to high-altitude adaption in multiple genera.^5–10^ Although the revealed genes vary greatly, their functions are always annotated to be involved in similar molecular pathways, including both DNA repairs and other physiological adaptations in response to alpine habitats.

The genus *Crucihimalaya* (belongs to Brassicaceae) contains 2–4 self-pollination diploid species with chromosome number of 2*n* = 16, mainly distributed in the high-altitude regions of the Qinghai–Tibet Plateau.^11^^,^^12^ The species in this genus were previously placed in the genera *Sisymbrium* or *Arabidopsis* because of their morphological similarity until the recent taxonomic revisions.^11^ Phylogenetic analyses based on sequence variations of the nuclear ribosomal internal transcribed spacer (ITS) region and other nuclear genes^13^ further supported the separation of this genus from *Sisymbrium* or *Arabidopsis*. The rough genome of one species from this genus, *C. himalaica*, was reported based on the Second-Generation Sequencer.^14^ Compared with the low-altitude genera *Capsella* and *Arabidopsis*, gene families related to disease resistance in this species were found to have significantly contracted while those to DNA repair and biquitin-mediated proteolysis expanded. In addition, genes related to reproductive processes were found to have experienced positive selection and/or functional loss in *C. himalaica*, although it remains unclear whether the identified genomic changes are common for all species of the genus occurring in the high altitudes or especially for *C. himalaica*. In addition, as the genome sequences of this species had not been assembled into chromosomes, the karyotype origin of the genus *Crucihimalaya* remains unresolved. Although the karyotype evolution in Brassicaceae is highly diverse, it can be traced to the recombination of two basic ancestral karyotypes.^15^^,^^16^ The chromosome-level genome sequence could be used to examine the karyotype origin of the genus from such ancestral karyotypes.^17^

In this study, we report a chromosome-level reference genome for another *Crucihimalaya* species, *C. lasiocarpa*, with a total size of 255.8 Mb and a scaffold N50 size of 31.9 Mb. Almost all protein-coding genes (99.13%) are anchored on eight chromosomes. In addition, we first examined the karyotype origin for the genus based on this chromosome-level genome sequence. Two other *Crucihimalaya* species without genomic resources are very similar to *C. lasiocarpa* and *C. himalaica*^11^^,^^12^ and these two species with available genomes likely represent the genetic diversity of the genus. Therefore, we further examined genomic evolution of the genus and how it was associated with alpine adaptation after conducting comparative genomic analyses of the two *Crucihimalaya* species. The renewed high-quality genome sequence for this genus will be highly useful for exploring the relative contributions of various genomic mechanisms in driving the adaptive evolution to high-altitude environments.

## 2. Materials and methods

### 2.1. Sample collection, DNA extraction and sequencing

**Figure 1.**
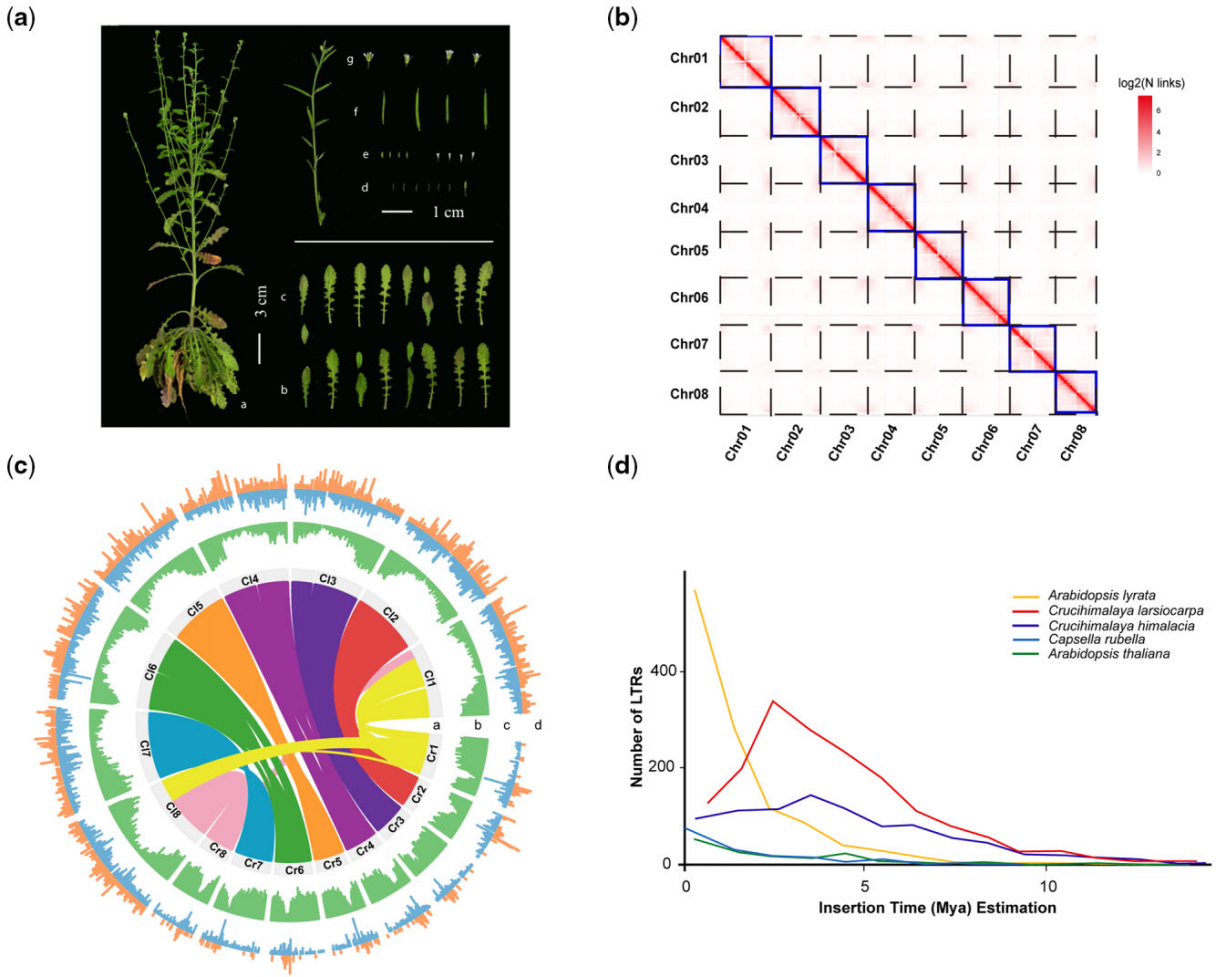
Summary of *Crucihimalaya lasiocarpa* genome assembly. (a) Photo of *C. lasiocarpa*: schematic representations of (i) aerial parts, (ii) adaxial surfaces of leaves, (iii) abaxial surfaces of leaves, (iv) stamens, (v) calyx and petal, (vi) fruit pods and (vii) flowers. (b) The Hi-C chromatin interaction map for the eight pseudochromosomes of *C. lasiocarpa*. (c) Genome comparison between *C. lasiocarpa* and *Cap. rubella*: (i) syntenic relationships between *C. lasiocarpa* and *Cap. rubella* genomes; (ii) gene density (window size = 100 kb, nonoverlapping); (iii) density distribution of *Copia* elements (window size = 100 kb, non-overlapping); and (iv) density distribution of *Gypsy* elements (window size = 100 kb, non-overlapping). (d) Number distribution and estimated insertion times of intact LTR retrotransposons.

Seeds of *C. lasiocarpa* were collected from Tibet, China (altitude 4,000 m, N 39.718°, E 91.120°) and germinated in the greenhouse (Fig. 1a). The whole steps of library construction and sequencing were performed at Grandomics Biotechnology Co., Ltd (Wuhan, China). The high-quality genomic DNA was extracted from fresh leaves of single individual by the Qiagen Genomic kit. Size selection was performed using the Blue-pippin system (Sage Science) to obtain DNA fragments from 10 to 50 kbp. The following library constructed using 1D ligation kit (SQK-LSK109) were sequenced on a PromethION platform (Oxford Nanopore Technologies, UK). Base calling was completed by Guppy v3.2.2 18 with default parameters. ONT reads with mean quality scores ≥ 7 (q7) were retained for the following genome assembly.

Furthermore, the paired-end Illumina libraries with insert sizes of 400 bp were prepared using the Illumina Genomic DNA Sample Preparation Kit, which were then sequenced on an Illumina NovaSeq platform (Beijing, Grandomics Biotechnology Co., Ltd). The filtered clean data was used for *k*-mer analysis and also the error correction. Furthermore, RNA-Seq reads of fresh leaf and root were generated for gene annotation using the MGI-2000 platform (Shenzhen, BGI Genomics Co., Ltd).

For Hi-C sequencing, fresh leaves from one plant were first fixed by formaldehyde. The fixed chromatins were then digested with DpnII restriction endonuclease.^18^ The digested nucleotide fragments were biotinylated with further proximity ligation to form chimeric circles.^19^^,^^20^ The DNA was further purified and sheared into fragments again for preparing the sequencing library. Finally, the chromatin conformation capture library was sequenced on an Illumina NovaSeq PE150 platform.

### 2.2. Genome assembly and chromosome construction

Before genome assembly, *k*-mer frequency distribution analysis was applied to estimate genome size and heterozygosity (size 21 bp in Illumina DNA short reads). A total of 37.72 Gb raw Nanopore long reads were corrected, assembled using NextDenovo v2.0-beta.1 (https://github.com/Nextomics/NextDenovo (24 January 2022, date last accessed)) with parameter of seed_cutoff = 38k, reads_cutoff = 1k. The preliminary assembly of *C. lasiocarpa* was followed by three rounds of polishing using NextPolish^21^ with both nanopore and short Illumina sequencing reads. Then, we mapped Hi-C data to the contigs using Juicer v1.6.2 pipeline^22^^,^^23^ and built primary scaffolds by the 3D-DNA v180922 with default parameters.^24^ Juicebox Assembly Tools v1.9.8 was used to visualize and manually curate the assembly.^25^ Afterwards, we processed another round of scaffolding by 3D-DNA v180922 to obtain the final pseudochromosomes. To assess the accuracy of the genome assembly, Illumina short reads were mapped against the genome using BWA v0.7.12-r1039 with default parameters.^26^ Benchmarking Universal Single-Copy Orthologous gene analysis (BUSCO) with the gene content of Embryophyta_odb10 were used to further evaluate the completeness of the assembled genome.^27^ LAI (LTR Assembly Index) scores were calculated using the LTR_retriever pipeline.^28^

### 2.3. Repeat annotation

Repetitive elements in *C. lasiocarpa* genome were identified by a combination method of homology-based and *de novo* strategies using RepeatModeler v1.0.11^29^ and RepeatMasker v4.0.7^30^ with default settings. LTRharvest v1.5.10^31^ and LTRFinder v1.06^32^ were used to detect full-length long terminal repeat retrotransposons (FL-LTR-RTs) in genome. The resulting outputs (.scn) from both analyses were fed into the LTR_retriever v1.9 programme^28^ to extract FL-LTR-RTs. The insertion time (T) for each LTR retrotransposons was calculated by the formula: *T* = *K*/2r using a substitution rate (*r*) of 7*10^−9^ substitutions per site per year, where *K* represented genetic distance.^33^

### 2.4. Gene prediction and function annotation

For accurate prediction of genes in the *C. lasiocarpa* genome, an integrated computational approach was adopted based on transcript mapping, homologous protein alignment and *ab initio* prediction. Illumina RNA-Seq raw reads were first processed using Trimmomatic v0.33^34^ to detect and remove adapter and low-quality bases (Phred quality score <20). The transcriptome assembly was then performed by Trinity v.2.6.6 using filtered reads.^35^ The assembled transcripts were further aligned to the assembled genome to carry out ORF prediction by PASA v2.1.0 pipeline.^36^ Protein sequences from *Aethionema rabicum*, *A. lyrata*, *Arabidopsis thaliana*, *Boechera stricta*, *Brassica rapa*, *Capsella grandiflora*, *Capsella rubella*, *Carica papaya*, *Leavenworthia alabamica*, and *Tarenaya hassleriana* (Supplementary Table S1) were aligned to the *C. lasiocarpa* genome using exonerate v2.4.0.^37^*Ab initio* gene prediction was carried out by Augustus v3.2.3 and GlimmerHMM v3.0.452 with parameter files generated by PASA self-trained gene models.^38^ Gene model evidence from the aforementioned results were integrated into a non-redundant set of gene annotations with EvidenceModeler v1.1.1.^39^ All predicted genes were analysed for functional domains and homologs by searching against the InterPro v32 using InterProScan^40^ and alignment to the integrated protein sequence databases including Swiss-Prot and TrEMBL.^41^ Subsequently, GO and KO annotation was analyses based on Blast2GO v2.5^42^ and KEGG pathway database.^43^

### 2.5. Genomic blocks identification

To better understand the evolutionary scenario of the *C. lasiocarpa* genome, we identified syntenic blocks in the *C. lasiocarpa* genome relative to the 22 genomic blocks (A–X) in ancestral crucifer karyotype (ACK).^15^^,^^44^ As genomic blocks in the ACK were defined using the *A. thaliana* gene IDs as start and end coordinates, homologous gene pairs were detected by BLASTP with a cut-off e value of 1e^−5^ of the predicted protein sequences against the *A. thaliana* proteome. Next, syntenic relationships were further determined using MCScanX^45^ and LAST v.946.^46^ Based on the collinearity in each genomic block against *A. thaliana*, we determined the corresponding boundaries and intervals of each block in *C. lasiocarpa* and renamed the pseudochromosomes. The genomic blocks in *Capsella rubella* were obtained through the same method applied in *C. lasiocarpa*. In order to eliminate false chromosomal rearrangements caused by assembly errors, the raw Hi-C reads of *C. lasiocarpa* were used to map to the whole genomic sequence of *Capsella rubella* using 3D-DNA v180922. Finally, we examined the order, orientation and contiguity of genes across potential adjacent segments, where inter-chromosomal rearrangements were inferred. The most parsimonious scenario of the fusion and fission events during genome evolution of *C. lasiocarpa* from its ancestral chromosomes was determined. The breakpoint regions were identified by pairwise genome alignment using LAST and MCScanX. By inferring putative homologous genes and collinear genes between *Capsella rubella* and *C. lasiocarpa*, we drew a homologous gene dot plot using WGDI.^47^

### 2.6. Phylogenetic and gene family analyses

Orthologous groups were identified using OrthoFinder v2.3.12^48^ by all-versus-all BLASTP alignments (E-value ≤ 1e^−5^) with protein sequences from *C. lasiocarpa, Crucihimalaya himalaica*, *Capsella rubella*, *A. thaliana*, *Eutrema heterophyllum*, *Eutrema yunnanense*, *Eutrema salsugineum* and *Aethionema arabicum* (Supplementary Table S2). We kept the longest transcripts of each gene model to eliminate redundancy caused by alternative splicing variations. Orthogroups with only one gene copy per species (Single-copy orthogroups) were collected, and aligned using Mafft v7.313 with globalpair G-INS-i strategy.^49^ The alignments of each single copy orthogroups were concatenated into a super-alignment. The super-alignments were then filtered by Gblocks v.0.91b^50^ to remove gap regions. Subsequently, phylogenetic trees were constructed by RAxML v8.2.11^51^ using the GTRGAMMA model and performed 100 bootstrap analyses to test the robustness of each branch.

Divergence time estimation was performed by MCMCTree in PAML v4.9 package,^52^ which implements the Markov chain Monte Carlo algorithms of Yang and Rannala.^53^ Analyses were run for 100,000 generations with a burn-in of 1,000 iterations. The calibrated molecular clock used to estimate divergence time (32–43 MYA between the *A. arabicum* and *A. thaliana*) was obtained from the TimeTree database.^54^ All MCMCTree calculations were run twice to ensure convergence.

We employed CAFE v4.2^55^ based on a Bayesian method to discover gene family contraction and expansion events using OrthoFinder results as input. The rooted and bifurcating tree from the phylogenetic analysis were used to time-calibrate the gene trees. To check the significance of contraction/expansion events at specific branches, we computed the branch-specific *P*-value and family-wide *P*-value with the Viterbi method for each orthogroup. Genes in significantly expanded families were then used for Gene Ontology enrichment analysis. The KOBAS software^56^ was also used to test the statistical enrichment of genes in KEGG pathways.

### 2.7. Identification of positively selected genes

To search for genes that evolved under positive selection (PSGs), single-copy gene families were extracted by OrthoFinder. Based on the non-synonymous to synonymous substitution ratio, the Branch-Site Model and Branch Model in CODEML from PAML package were used to detect selection with the ancestral branch leading to the *C. lasiocarpa* and *C. himalaica* set as foreground branch. For the Branch-Site Model, the *χ*^2^ test was conducted for each orthologous to assess statistical significance of Model A and Model A null. Finally, Bayes empirical Bayes (BEB) analysis^57^ was used to identify gene with positively selected sites of posterior probabilities greater than or equal to 0.99. For Branch Model, we ran the one-ratio branch model (null model which assumes that all branches have been evolving at the same rate) and the multi-ratio model (alternative model which supposing foreground branch to evolve under a different rate) to estimate the *dN*/*dS* ratio in each orthologs. A likelihood ratio test (LRT) with df = 1 was employed to discriminate between the null model and the alternative model.^58^ Genes with a *P*-value <0.05 and a higher *dN*/*dS* value in the foreground than the background branches were regarded as positively selected genes in the foreground.^59^

### 2.8. S-locus structure and self-fertilization

Known that loss of function at the S-locus is the reason for self-fertilization of *C. himalaica* and the genus Crucihimalaya is self-compatible,^14^ we searched S-haplogroups sequences in the *C. lasiocarpa* to explore if same mutations could be in *C. lasiocarpa*. The published S-haplogroups sequences of *Crucihimalaya himalaica*, *Capsella grandiflora*, *A. thaliana*, *Arabidopsis halleri*, *A. lyrata* and *B. rapa* were retrieved from NCBI and used as query in BLASTp searches against the genome assembly of *C. lasiocarpa*. Via searches, we manually annotated the candidate homologous gene of S locus *SCR* gene and the flanking genes U-box gene. Intriguingly, there was a candidate protein hit, which matched ARK3 and SRK. Due to the confusing role of candidate ARK3 and SRK protein, the SRK and ARK3 protein of *Arabidopsis* were downloaded and aligned by MAFFT. Then, a maximum likelihood tree both including SRK and ARK3 was constructed using RaxML. All candidate proteins of S-locus were further confirmed by hmmsearch against the Pfam database.

## 3. Results

### 3.1. Genome assembly and gene annotation

The genome size of *C. lasiocarpa* is estimated at ∼253.6 Mb, with a heterozygosity rate of 0.04% based on *k*-mer analysis (Supplementary Fig. S1). To obtain a high-quality, chromosome-scale genome assembly, we produced 139× coverage of Oxford Nanopore long-reads sequencing data (37.72 Gb), 53× coverage of paired-end Illumina short reads sequencing data (18.00 Gb) and 104× coverage of paired-end Hi-C reads (54.99 Gb) (Supplementary Tables S3 and S4). Firstly, the Oxford Nanopore long reads and Illumina short reads were used for primary assembly, comparison and error correction, which finally produced a total of 255.82 Mb genome assembly with an average contig N50 of 14.98 Mb, close to the estimated genome size of 253.6 Mb (Supplementary Fig. S1). With the assistance of Hi-C reads, we then anchored contigs into eight pesudochromosomes using 3D-DNA pipeline (Figure 1b). Compared to the previously published *C. himalaica* genome assembly based on Illumina short-read technology that produced an assembly of 583 scaffolds (3,983 contigs), with a scaffold N50 of just 2.0 Mb (contig N50 = 136.3 Kb), the final genome assembly of *C. lasiocarpa* exhibited a total size of 255.81 Mb, including 20 scaffolds with a 31.9 Mb scaffold N50 size, and the largest scaffold size was 35.0 Mb. The gap rate of the assembled *C. lasiocarpa* sequences was estimated to be only 0.003%, while it was 1.63% for the *C. himalaica* genome assembly (Table 1). We further evaluated the completeness of the assembled genome, and found high completeness (99.6% of *C. lasiocarpa* and 99.7% of *C. himalaica*) rate of both assemblies as evidenced by BUSCO analysis^60^ (Supplementary Fig. S2). The long terminal repeat (LTR) Assembly Index (LAI), which evaluates the contiguity of intergenic and repetitive regions of genome assemblies based on the intactness of LTR retrotransposons (LTR-RTs),^61^ was 17.64 for *C. lasiocarpa* genome assembly, which was substantially higher than the LAI values obtained for the *C. himalaica* genome and other relatives under comparison (Supplementary Fig. S3).

**Table 1:** Statistics for *C. lasiocarpa* and *C. himalaica* genome assemblies.

Species	*Crucihimalaya lasiocarpa*	*Crucihimalaya himalaica*
Sequencing platform	ONT PromethION	Illumina HiSeq 2500
Assembly size (bp)	255,812,582	234,722,603
GC %	36.49	36.38
Number of scaffolds	20	583
Longest scaffold (bp)	35,013,560	8,343,586
Scaffold N50 size (bp)	31,983,042	2,088,603
Scaffold N90 size (bp)	27,759,296	470,087
Number of Scaffold N50	4	34
Number of Scaffold N90	8	129
Number of contigs	58	3,983
Longest contig (bp)	21,500,254	1,756,581
Contig N50 size (bp)	14,980,479	136,392
Contig N90 size (bp)	11,549,935	32,421
Number of Contig N50	8	406
Number of Contig N90	15	1711
Gap %	0.003	1.63
Number of genes	24,169	27,019

Based on a combination of *de novo* prediction, homology searching, and transcriptome-based approaches, we annotated 24,169 protein-coding genes in the *C. lasiocarpa* genome. Around 99.13% of the genes (23,960 out of 24,169) were anchored to eight chromosomes while only 0.86% (208 out of 24,169) were left on the un-anchored scaffolds. The average gene length, coding sequence length and an average exon number were estimated to be 2,582 base pairs (bp), 236 bp and 5.53 exons, respectively (Supplementary Fig. S4). These protein-coding genes were further functionally annotated based on Swiss-Prot, InterPro, GO and KEGG Pathway databases (Supplementary Table S5). The average gene length, coding sequence length and an average exon number were estimated to be 2,582 base pairs (bp), 236 bp and 5.53 exons, respectively (Supplementary Fig. S3). In contrast to the many short-length genes identified in *C. himalaica*,^14^ the gene lengths of *C. lasiocarpa* are normally distributed as those of *A. thaliana*. Furthermore, the completeness of the gene annotation was also assessed using BUSCO, and the result revealed a higher proportion of complete single-copy orthologs in the genome assembly of *C. lasiocarpa* (97.2%) compared to that in *C. himalaica* (96%) (Supplementary Table S6), suggesting that *C. lasiocarpa* genome seems to have a high gene-annotation quality.

### 3.2. Evolutionary origin of repeat elements in *Crucihimalaya lasiocarpa*

Based on *de novo* and homology prediction approaches, a total of 134.5 Mb (52.58%) of the assembled *C. lasiocarpa* genome was identified as repeat regions with LTR elements being identified as the major class (27.01%) (Supplementary Table S7). The proportion of repetitive elements near the centromere is higher than other regions (Fig. 1c). *Crucihimalayalasiocarpa* contains a higher proportion of repeat elements than *C. himalacia*, *A. thaliana* and *Capsella rubella.*^62^^,^^63^ We did not find that *C. lasiocarpa* undergoes an additional species-specific WGD event compared with *C. lasiocarpa*, *A. thaliana* and *Capsella rubella* based on the distribution of synonymous substitutions per synonymous site (Ks) among paralogous genes within the genome (Supplementary Fig. S5).

We further explored the evolutionary dynamics of the intact long terminal repeat retrotransposons (LTR-RTs) in the genomes of *C. lasiocarpa* and other three closely related species. A total of 1,719,930,178 and 1,166 LTR-RTs were identified in *C. lasiocarpa*, *C. himalacia*, *Capsella rubella* and *A. lyrata* genomes, respectively. LTR retrotransposons in the genome of *C. lasiocarpa* had recently undergone a rapid proliferation ∼2.5 million years ago (Fig. 1d). As the insertion of an LTR into a new position in the genome might lead to alternative splicing of a particular transcript through various mechanisms, resulting in new genetic and phenotypic variations with potential adaptive significance.^64^ Compared with *C. himalacia*, we found that 152 orthologous genes in *C. lasiocarpa* contained specific insertion of *Gypsy* or *Copia* element. Further analysis revealed that these genes exhibited significantly higher protein evolutionary rates (*K*a/*K*s) compared with the genomic background (Supplementary Fig. S6).

### 3.3. Karyotype origin of *Crucihimalaya lasiocarpa*

**Figure 2.**
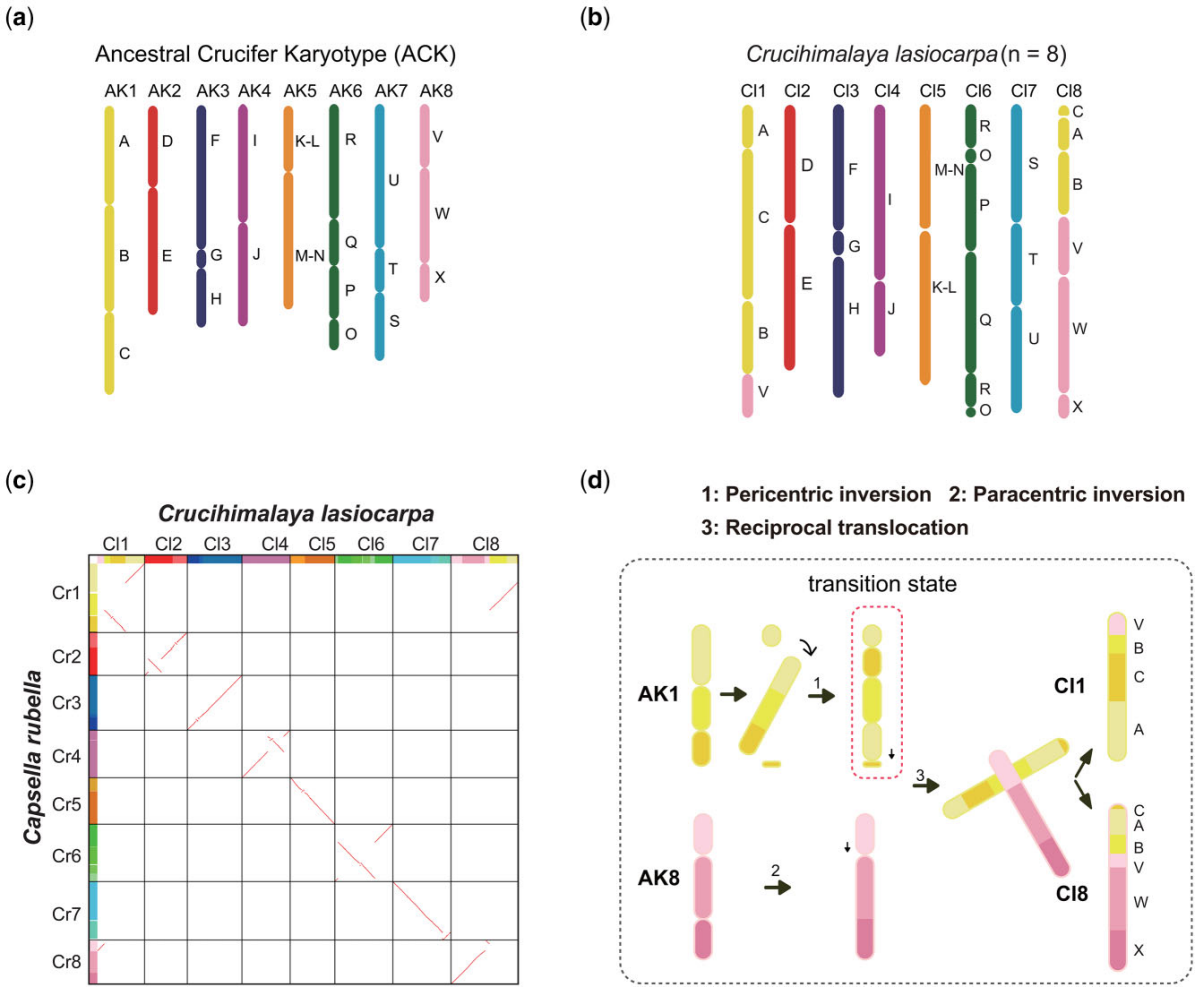
Ancestral crucifer karyotype and *Crucihimalaya lasiocarpa* genomic blocks. (a) The ancestral genomes ACK comprising 22 ancestral GBs. (b) Twenty-two GBs and their positions within the *C. lasiocarpa* genome. (c) Syntenic dot plot of *C. lasiocarpa* and *Cap. rubella*. The dot plot was generated using programme WGDI. Assignment to genomic blocks is given on the left for *Capsella* and above for *Crucihimalaya*. Syntenic genes are coloured by Ks values. Only gene pairs with Ks value lower than 0.6 are retained. (d) Chromosomal rearrangements illustrating the origin of *Crucihimalaya* genome (*n* = 8) from ACK-like genome (*n* = 8) are showed.

Chromosomal structural variations play a critical role in phenotypic variation and environmental adaptation.^65^ Using comparative chromosome painting (CCP) techniques, Ancestral Crucifer Karyotype (ACK) for the family Brassiaceae with eight chromosomes (*n* = 8) with 22 conserved genomic blocks (GBs, A to X) were suggested^66^ (Fig. 2a). These defined GBs and the changed compositions from the ACK were used to examine karyotype evolution in other genera and species. As the karyotype of *Capsella rubella* was suggested to be similar to the ACK of the family Brassicaceae,^63^ we compared karyotypes of *C. lasiocarpa* and *Capsella rubella* using previously reported methods (Supplementary Figs S7 and S8).^17^^,^^67^ We compared the genomic blocks of *C. lasiocarpa* with inferred ancestral karyotypes, including the ACK (*n* = 8), proto-calepineae karyotype (PCK; *n* = 7) and translocated PCK (tPCK; *n* = 7). The karyotype of *C. lasiocarpa* was inferred to evolve from the ACK. The *C. lasiocarpa* karyotype comprises six relatively conserved chromosomes (CL2, 3, 4, 5, 6 and 7) and two chromosomes with structural variations occurring (reciprocal translocation and inversion) (Fig. 2b).

The inversion fragments in CL2, 3, 4, 5 and 7 chromosomes are small and do not span GBs boundary, so the order between the GBs in the chromosomes remained unchanged. CL6 possesses a pericentric inversion with a length of ∼25M. This inversion spans the four GBs of CL6 (R, Q, P and O) as the longest inversion across the whole genome (Supplementary Fig. S9). The Hi-C interaction heatmap showed that this chromosomal rearrangement was not caused by assembly errors (Supplementary Fig. S9). The majority of these events occurred in the pericentromeric regions of the *C. lasiocarpa* chromosomes (Fig. 2c and d).

### 3.4. Phylogenetic analyses and expansion and contraction of gene families

**Figure 3.**
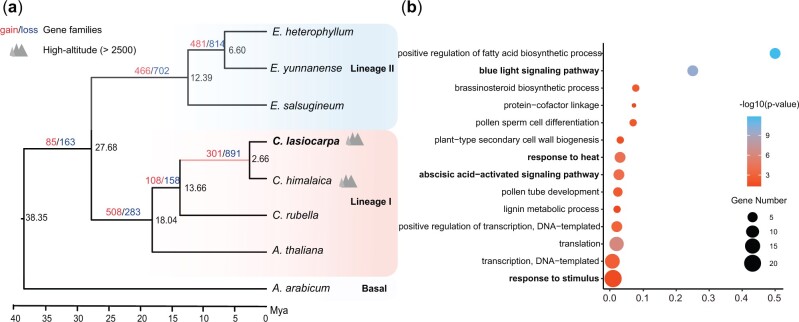
Phylogenetic analysis of the *Crucihimalaya lasiocarpa* genome. (a) The phylogenetic placement of *C. lasiocarpa*, divergence time (million years ago, MYA, black), gene family expansions (red) and contractions (blue) are displayed on a maximum likelihood (ML) tree constructed from 5,993 shared single-copy gene families. (b) Gene ontology (GO) enrichment of significantly expanded gene family of high-altitude clade (highlighted by pink) in a. The colour of circles represents the statistical significance of enriched GO terms. The size of the circles represents the number of genes in a GO term.

A total 5,993 single-copy gene families were used to construct a maximum-likelihood phylogenetic tree for *C. lasiocarpa* and closely related species (Fig. 3a). The phylogenetic tree showed that the *C. lasiocarpa* was sister to *C. himalaica*, which diverged about 2.66 million years ago (MYA). The *C. lasiocarpa*–*C. himalaica* clade diverged from *Capsella rubella* ∼13.66 MYA.

Adaptation is greatly favoured by origination of new genes.^68^ Gene duplication offers a rapid way to produce new genes.^69^ We found 301 gene families expanded and 891 gene families contracted in the clade comprising two alpine species of the genus *Crucihimalayam*, *C. lasiocarpa* and *C. himalaica* (Fig. 3a). A total of 56 gene families were significantly expanded (*P*-value < 0.05), which are enriched for ‘blue light signalling pathway’, ‘response to stimulus’ and ‘abscisic acid-activated signalling pathway’ (Fig. 3b). These pathways have long been proven to be associated with resisting strong UV radiation and tolerating low temperature.^70^ We also found that some ubiquitin-conjugating gene families expanded in these species (Supplementary Table S8). Some gene families found functionally involved in biotic stress significantly contracted, including those related to camalexin biosynthesis, farnesene biosynthesis and Indole-3-acetate biosynthesis (Supplementary Table S9) which were found as special phytoalexin in response to bacterial and fungal pathogens^71^^,^^72^ and defensing against nematodes.^73^

### 3.5. Identification of candidate genes related to High-Altitude adaptation of the genus

**Figure 4.**
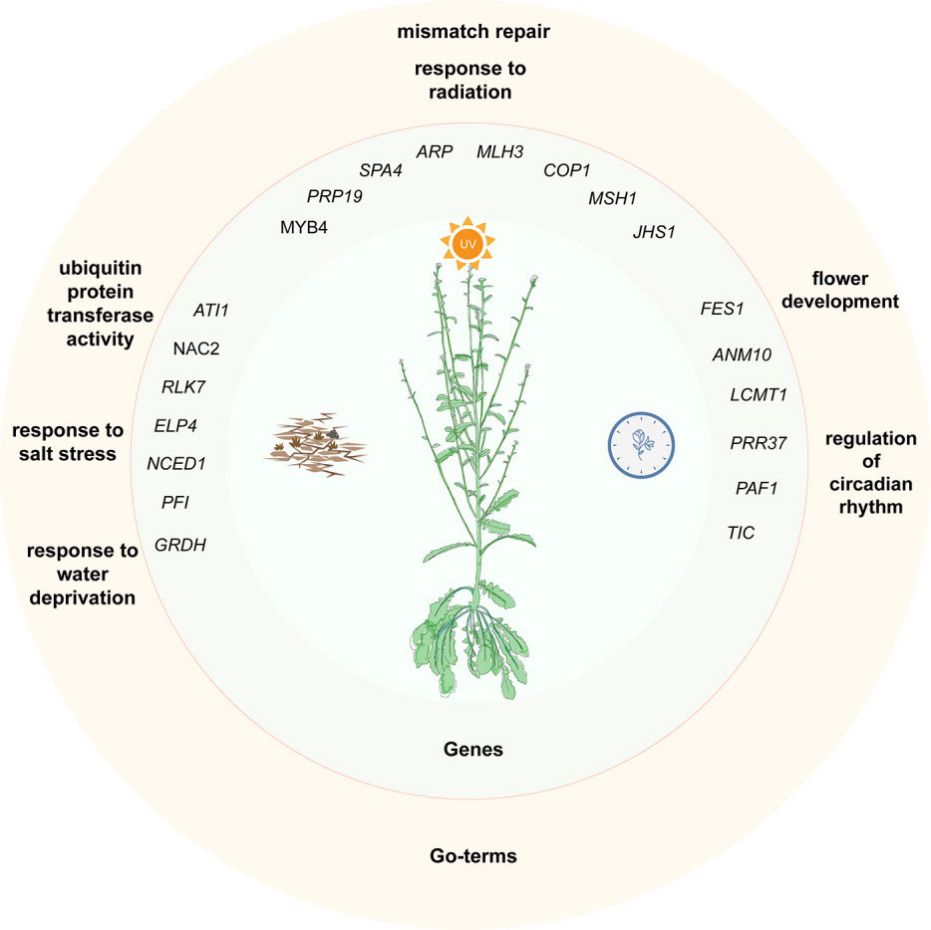
Functional adaptation of the positively selected genes (PSGs) of the genus *Crucihimalaya* to the high-altitude habitats. The outer cream-coloured circle shows examples of enriched biological process GO-terms. The inner light grey circle shows examples of candidate positive selected genes.

Stresses may lead to positive selections of the related genes.^74^^,^^75^ We identified positively selected genes (PSGs) for the clade comprising *C. lasiocarpa* and *C. himalaica*. Using Branch Model and Branch-Site Model of codeml in the PAML package, we totally identified 403 PSGs (Supplementary Table S10). The functions of the significantly positively selected genes (PSGs) were associated with stress tolerance and alpine survival (Fig. 4 and Supplementary Table S10). For example, the gene *HOS15* was found to be involved in resisting cold stress in plants through mediating deacetylation of histone.^76^ Both genes *MLH3* and *MSH1* play an important role in repairing DNA mismatches and correcting insertion-deletion loops,^73^ while *RLK7* is an important transcription factor to regulate resistances to abiotic stress in plants.^73^

### 3.6. Self-compatibility and S-locus structure of *Crucihimalaya lasiocarpa*

Self-pollination and self-compatibility (SC) in Brassiaceae arise through functional loss or disappearance of the self-incompatibility (SI) genes, including the stigma-expressed receptor kinase (*SRK*) gene at the S locus and the other pollen-expressed cysteine-rich (*SCR/SP11*) gene.^73–76^ SI species may be vulnerable to pollen limitation due to the lack of pollinators under typical high-altitude and harsh conditions. However, species can achieve optimal reproductive fitness through self-pollination.^77^ Using S-locus sequences from the close relatives to search against the *C. lasiocarpa* genome, we identified the SCR gene in our species. The *SCR* gene of *C. himalaica* had two mutations across the eight critical conserved cysteines.^14^ We also identified these two mutations in *C. lasiocarpa*, which is critical to maintain structural and functional integrity of the SCR protein^73–76^ (Supplementary Fig. S10). Using the same way, we also identified the likely *SRK* gene in *C. lasiocarpa*. Both *SRK* and *ARK3* belong to the plant receptor-like/Pelle kinase (RLKs) family.^78^ We used the *SRK* and *ARK3* sequences from *Arabidopsis* and other species to search for the corresponding homologous sequences. We constructed the maximum likelihood tree using all homologous sequences from two *Crucihimalaya* species and other species. We found that all identified homologous sequences from *Crucihimalaya* were clustered with the *ARK3* genes of the other species and none was clustered with the *SRK* genes (Supplementary Fig. S11).

## 4. Discussion

Here, we describe a chromosomal-scale genome for an alpine plant *C. lasiocarpa* in the QTP obtained by integrating data from ONT, Hi-C and Illumina platforms. The genome assembly of *C. lasiocarpa* exhibited a total size of 255.8 Mb, and it is the first chromosomal-scale genome in the genus *Crucihimalaya*. All of our statistics indicates that the genome assembly we generated was of high completeness and continuity, ensuring the reliability of our subsequent comparative genomic analyses. Compared with previously published genome *C. himalaica* with contig N50 of 136 kb and scaffold N50 of 2 Mb, the present *C. lasiocarpa* genome has highly improved contig N50 (14 Mb) and scaffold N50 (31 Mb). Our scaffolding with Hi-C further facilitated the accurate assignment of all scaffolds to chromosomal positions. The ONT-based assembly also annotated more complete repetitive sequences than the Illumina-based assembly.^79^ Therefore, the centromere region of the *C. lasiocarpa* genome seem to be more accurate and complete than that of *C. himalaica* (Supplementary Fig. S12). We identified substantially more TEs (especially intact LTR-RTs) in *C. lasiocarpa* than *C. himalaica*. The rapid proliferation of these repetitive elements and especially those inserted around genic regions in *C. lasiocarpa* may play a key role in promoting its genome evolution and species divergence from the congener *C. himalacia.*^14^

The karyotype of the genus *Capsella* is hypothesized to be similar to the inferred ancestral ACK karyotype (*n* = 8).^66^ Compared with this ACK karotype, the karyotype of our studied species *C. lasiocarpa* (*n* = 8) is relatively conserved because five chromosomes (Cl2, 3, 4, 5 and 7) remain stable while the other three show chromosomal structural variations, including two reciprocal translocation, two chromosome fusions and three inversions (Fig. 2d and Supplementary Fig. S9), which may have occurred since the divergence of *Crucihimalaya* from *Capsella* ∼11 million years ago (MYA).^80–82^ Another congener of the genus *Crucihimalaya, C. wallichii*, was also found to have a similar pericentric inversion as in *C. lasiocarpa* for the first chromosome (Supplementary Fig. S13).^82^ These chromosomal variations may be common for various genus or specific to each genus or species, since another inversion of Cl8 in *C. lasiocarpa* (Supplementary Fig. S13) is very similar to the V genomic block in the eighth chromosome of *Transberingia bursifolia* (Brassiaceae).^82^ This ‘rare genomic changes’^83^ may suggest phylogenetic relatedness and common ancestry, although the phylogenetic relationship between *Crucihimalaya* and *Transberingia* is unclear. Therefore, parsimonious karyotype evolution of this species (or the genus *Crucihimalaya*) may involve both fusion and fission events during reciprocal translocation and inversions (Fig. 2D). The fragments of AB genomic block in AK1 and V genomic block in AK8 broke off from the corresponding chromosomes and swapped places, and further formed chromosomes CL1 and CL8 in *C. lasiocarpa*. Future studies may still need to explore whether such a similar chromosomal rearrangement is derived from paralleling evolution or because they share the same common ancestor.

Self-pollination and SC through functional loss of the SI genes remains a common reproductive assurance for alpine plants when pollinators become scarce.^77^ The genus *Crucihimalaya* are self-compatible while most genera of the Brassicaceae are outcrossing with strong SI.^84^^,^^85^ In this family, SI was certified to be determined by sequence variation at a highly polymorphic S locus with *SRK* and *SCR/SP11* genes.^78^ The SRK protein performs as the receptor for SCR to distinguish the ‘non-self’ pollen during SI response. Self-compatibility is achieved usually when SRK receptors could not detect SCR ligands. Our study of *C. lasiocarpa* and the previous investigation of *C. himalacia*^14^ suggested that *SRK* genes seemed to have been lost for the genus *Crucihimalaya* and the *SCR* gene also showed the relaxed selection with two mutations that disrupt the normal function of this gene. Therefore, these two evolutionary events may together lead to the self-pollination shift of the genus. In addition, the identified positively selected genes (PSGs) for the genus are involved in DNA repair, coldness- and drought-response and reproductive processes.^14^ We also found the significantly expanded gene families involved in adaptation of alpine habitats. All of these results together suggest that the ancestor of *Crucihimalaya* might have developed such special genomic characters to adapt to the high-altitude QTP before they diversified into these two species. It remains unclear whether these two species diverged with chromosomal structural variations and what type of genes played a key role during their divergence. In order to address these questions, the chromosomal-scale genome sequence of *C. himalaica* needs to be assembled and population genomic data of these two species are expected to obtain in the future. In summary, the chromosomal-scale genome sequence of *C. lasiocarpa* was reported here for the first time. We also clarified the karyotype evolution for the genus of *Crucihimalaya.* Combined with the previously reported genome sequence for *C. himalaica*, we found that many genomic changes related to alpine adaptation might have occurred before the divergence of the two species.

## Supplementary data

Supplementary data are available at *DNARES* online.

## Supplementary Material

dsac004_Supplementary_Data
