# Supplementary material for: A chromosome-level genome assembly of an alpine plant Crucihimalaya lasiocarpa provides insights into high-altitude adaptation
Source: DNA Res. 2022 Jan 29;29(1):dsac004. doi: 10.1093/dnares/dsac004 (PMC8801980; doi:10.1093/dnares/dsac004)
Supplement: dsac004_Supplementary_Data [file dsac004_supplementary_data.docx]

**Supplementary Figures and Tables**

**Supplementary figures**


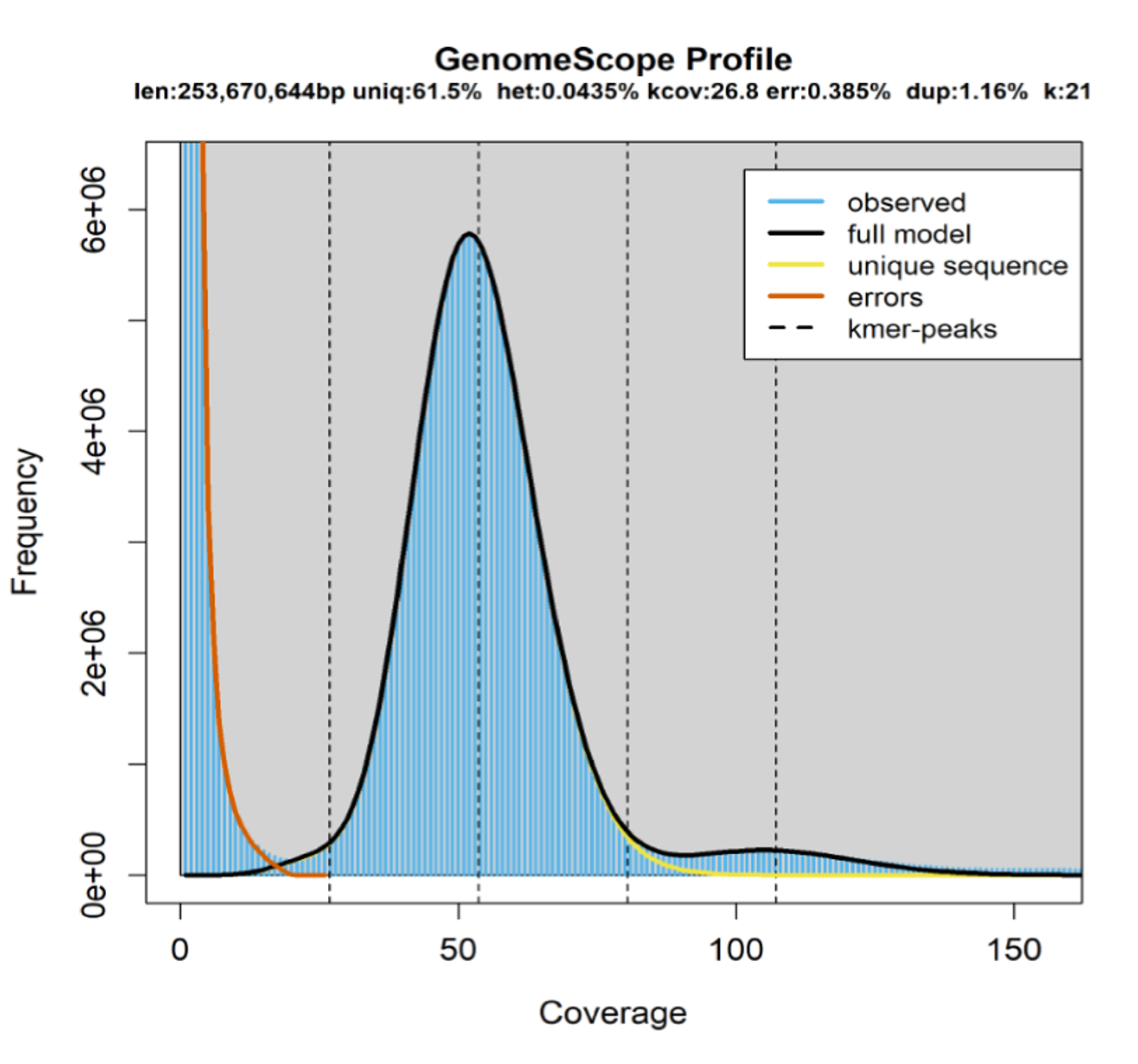


**Supplementary Figure 1. The *K*-mer analysis used to estimate *C. lasiocarpa* genome size.** The frequency of 21-mers were shown representing 21 bp sequences within reads (after filtering) from the clean reads of short-insert size libraries (250bp). The genome size of *C. lasiocarpa* was estimated to be 253 Mb.


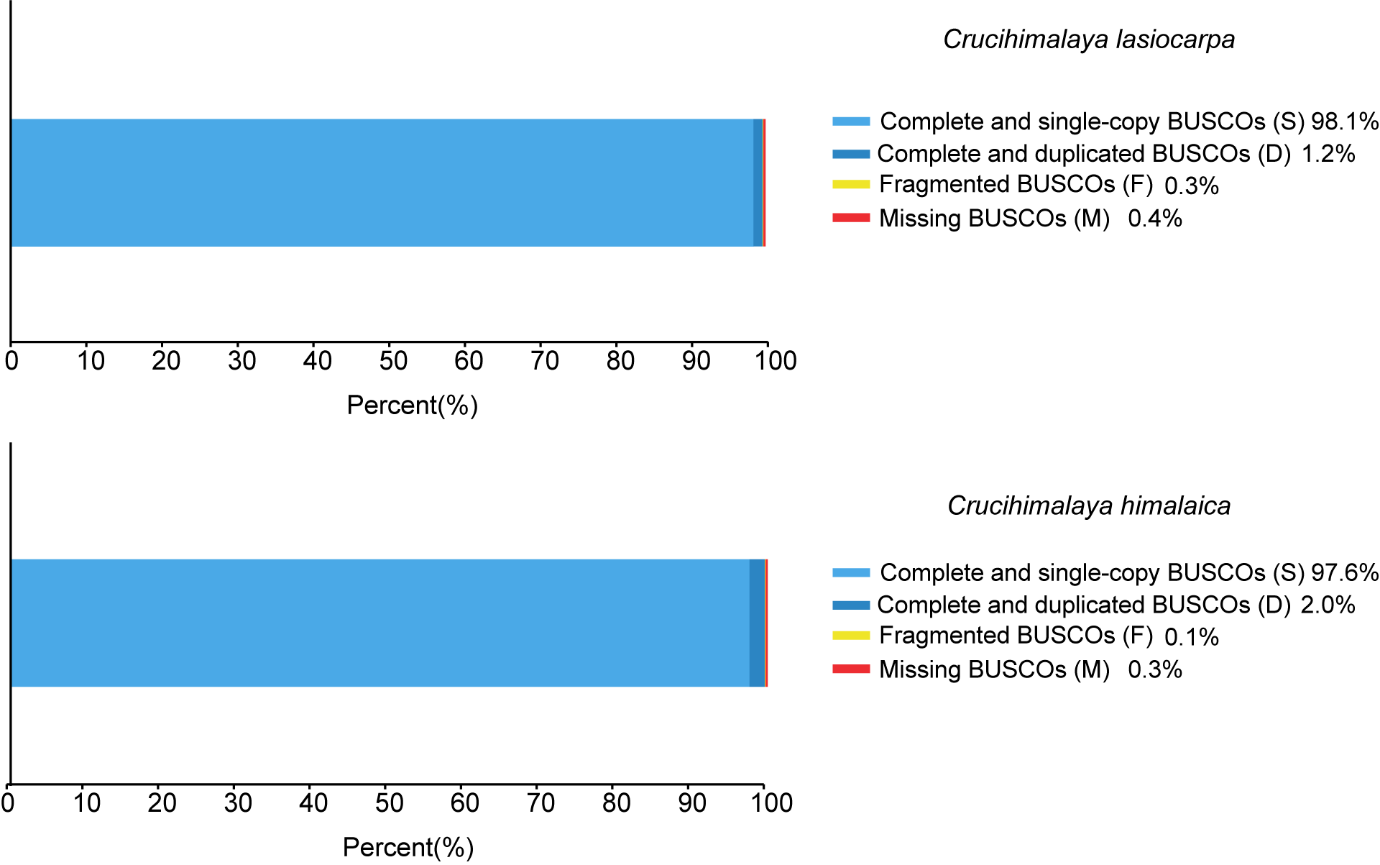


**Supplementary Figure 2. Completeness of conserved gene models.** Percentage of conserved genes in the plant kingdom (assessed by BUSCO) that can be found in the *C. lasiocarpa* and *C. himalaica* genomes.


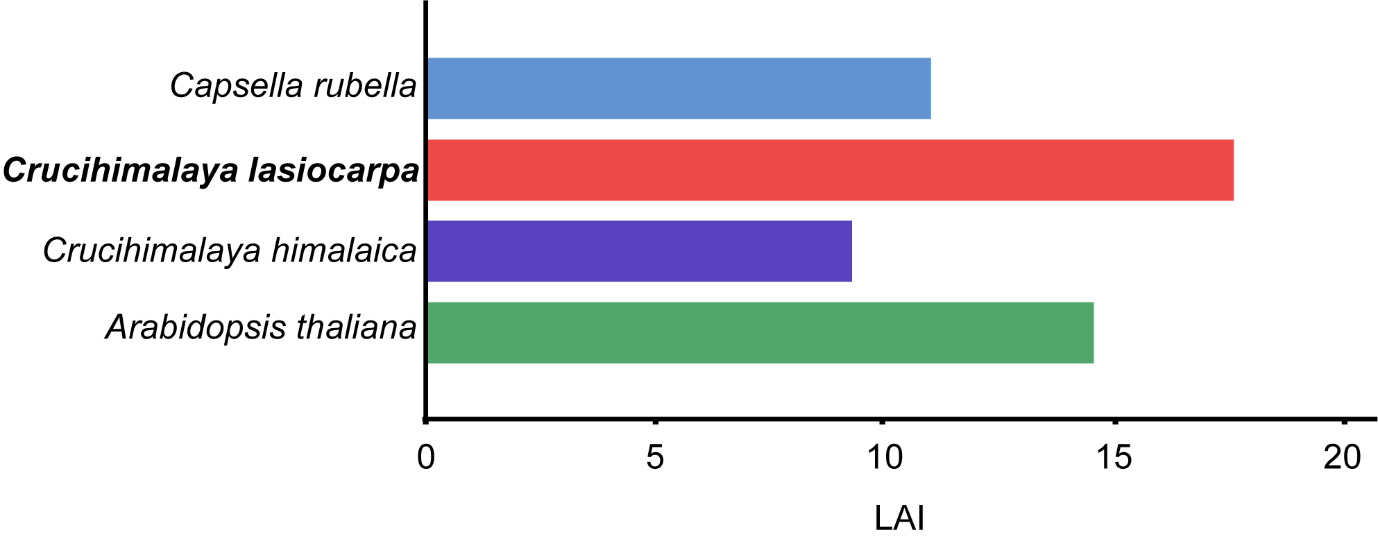


**Supplementary Figure 3.** **The long terminal repeat (LTR) Assembly Index (LAI) values of *C. lasiocarpa* and other Brassicaceae species.**


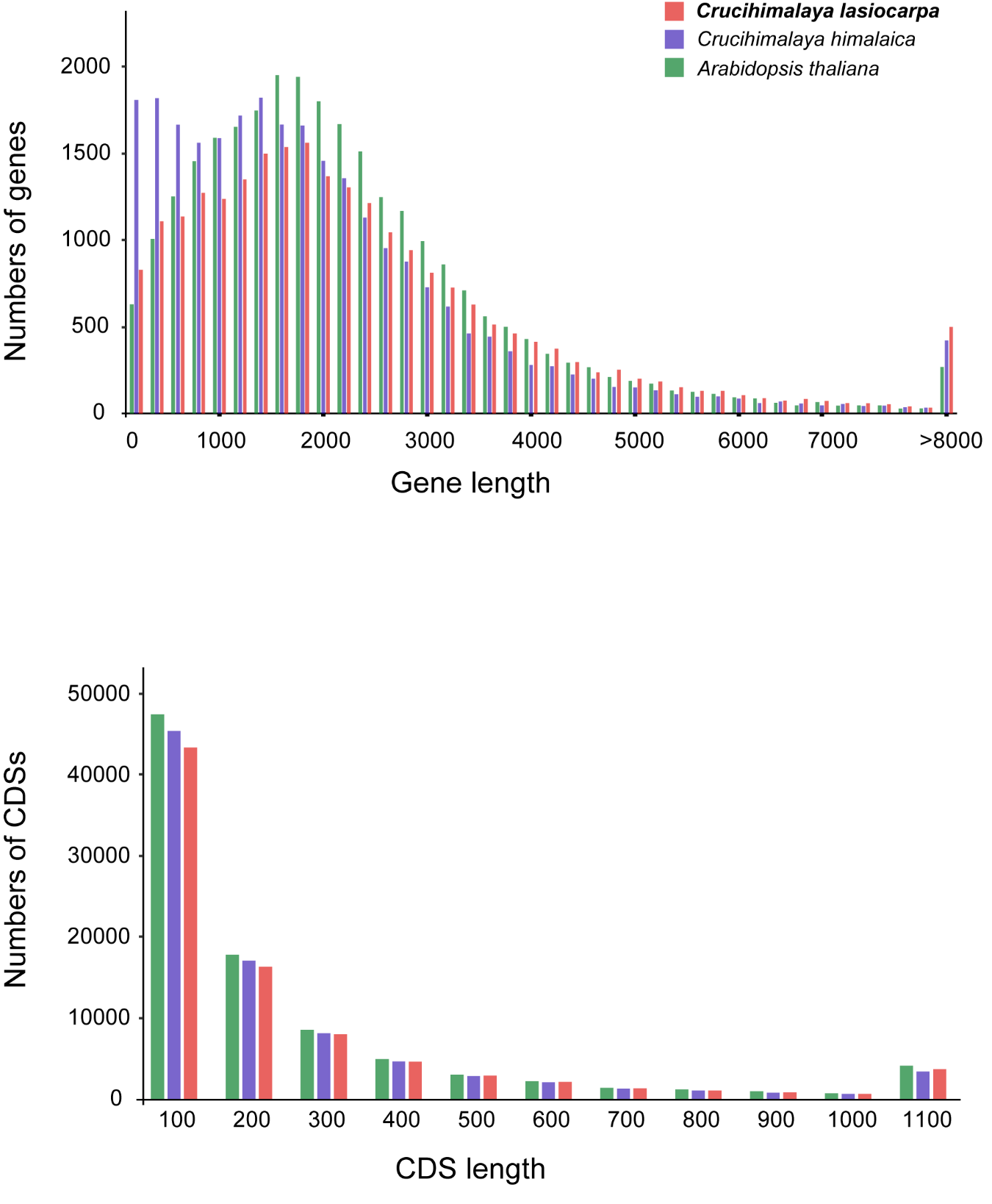


**Supplementary Figure 4. Gene model annotation statistics.** Length distribution of gene, CDS annotated in the *C. lasiocarpa* genome.


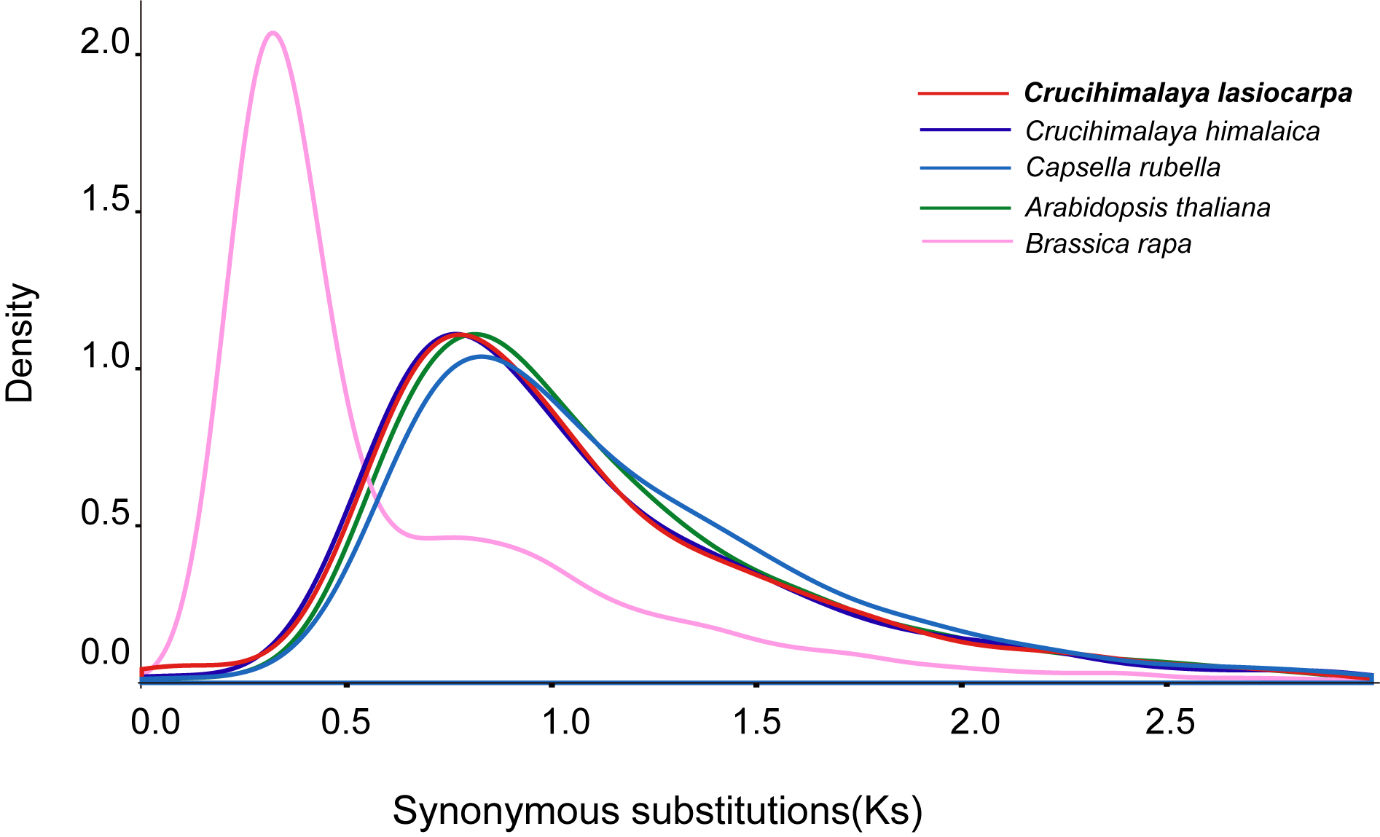


**Supplementary Figure 5.** **The Ks values of *C. lasiocarpa* and other Brassicaceae species.**

**
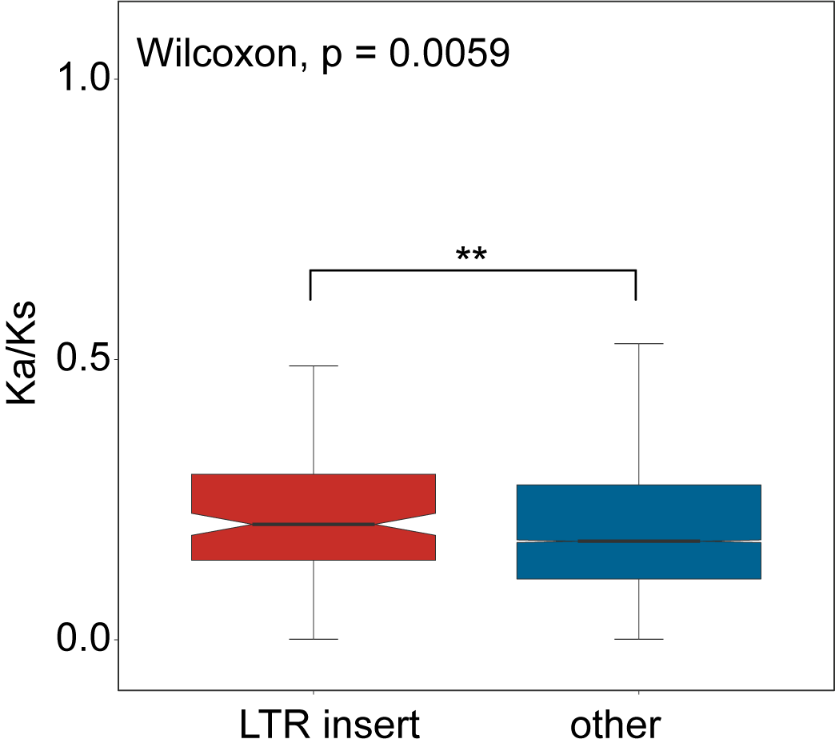
**

**Supplementary Figure 6. Ka/Ks distribution of orthologous genes between *Cap. rubella* and *C. lasiocarpa*.** The genes with specific insertion of *Gypsy* elements in *C. lasiocarpa* showed Ka/Ks values significantly higher than the other genes (significance tested by Wilcoxon method with p-value = 0.0059 < 0.05).


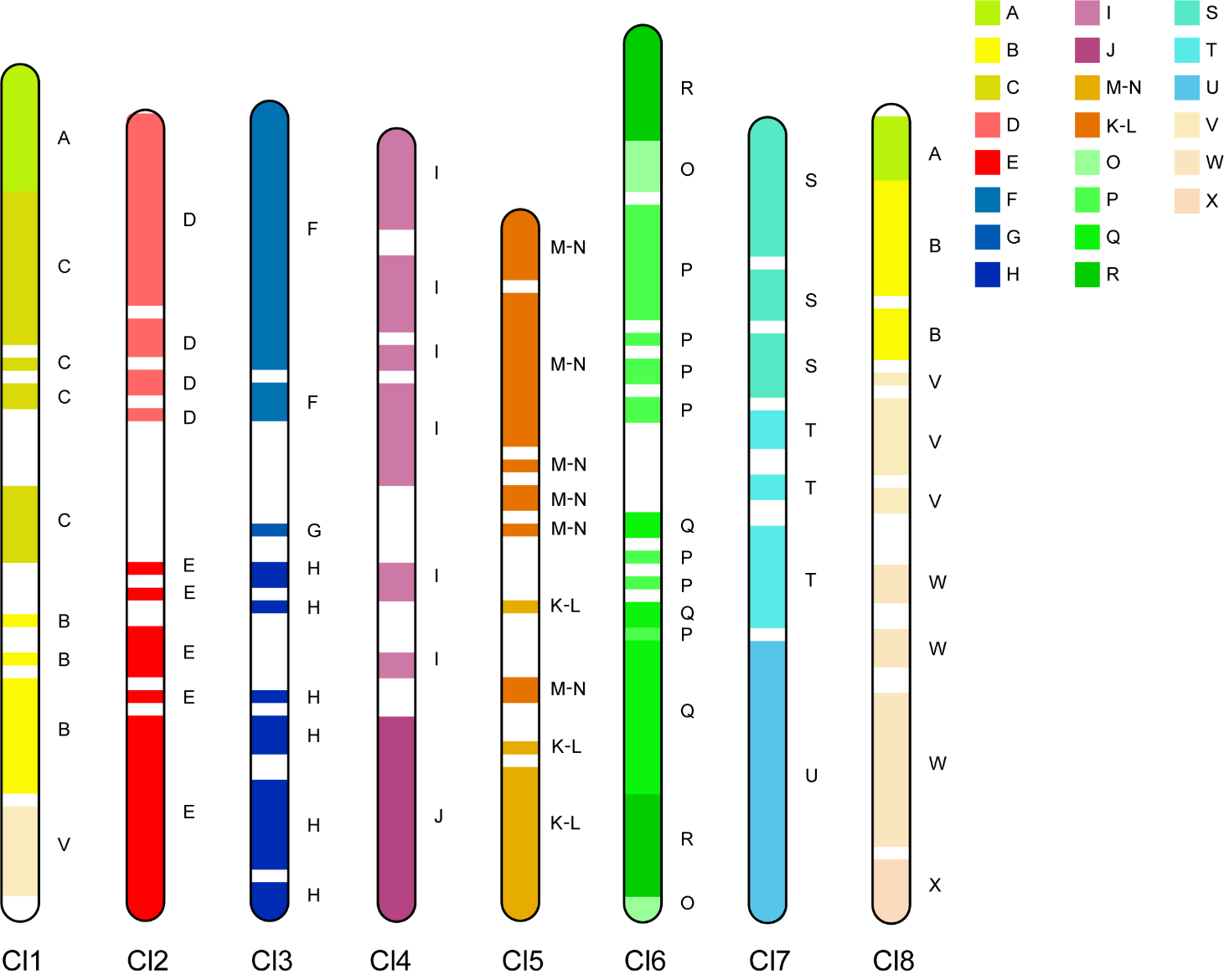


**Supplementary Figure 7. Genomic structure of *C. lasiocarpa*.** A reconstruction of the genome structure of *C. lasiocarpa* based on syntenic relationship with *A. thaliana*.


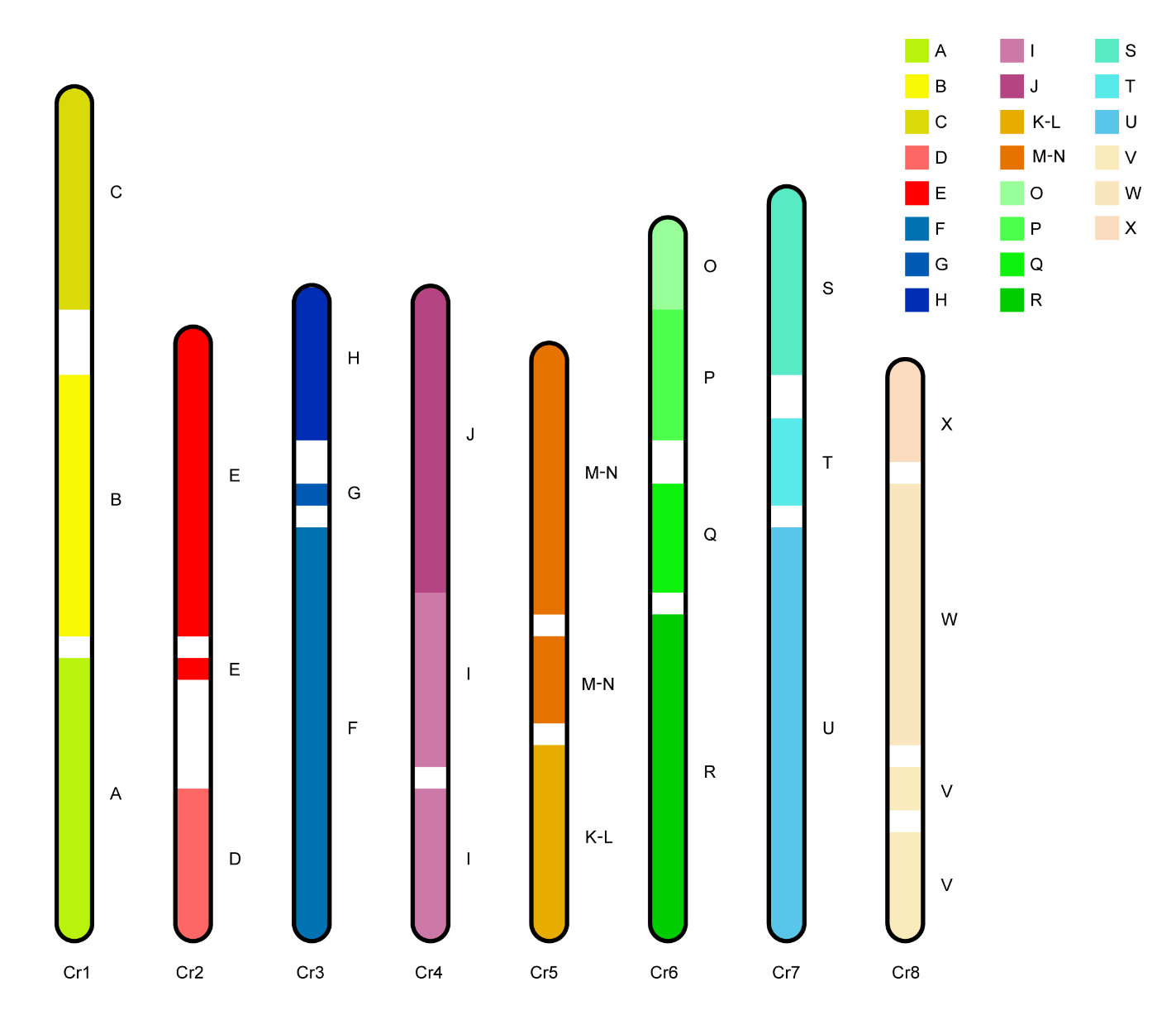


**Supplementary Figure 8. Genomic structure of *Cap. rubella*.** A reconstruction of the genome structure of *Cap. rubella* based on syntenic relationship with *A. thaliana*.


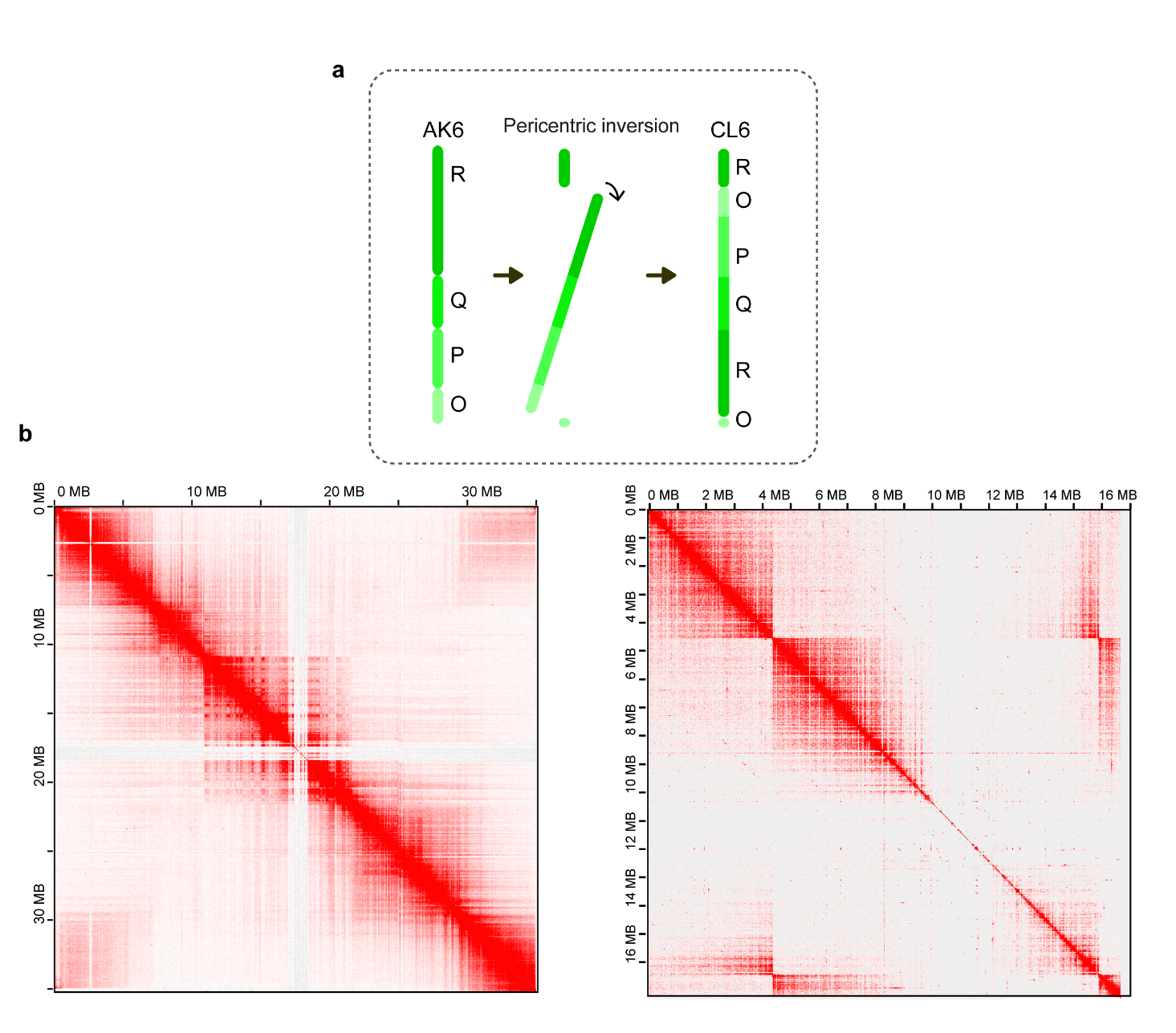


**Supplementary Figure 9. Chromosomal rearrangement of CL6 from ACK-like genome. A.** Schematic diagram of inferred most parsimonious scenario of CL6 chromosomal rearrangements. **B.** The Hi-C chromatin interaction map of CL6 on the left and the hic interaction heat map on the right generated by raw hic reads of *C. lasiocarpa* mapped to genome of *Cap. rubella*.


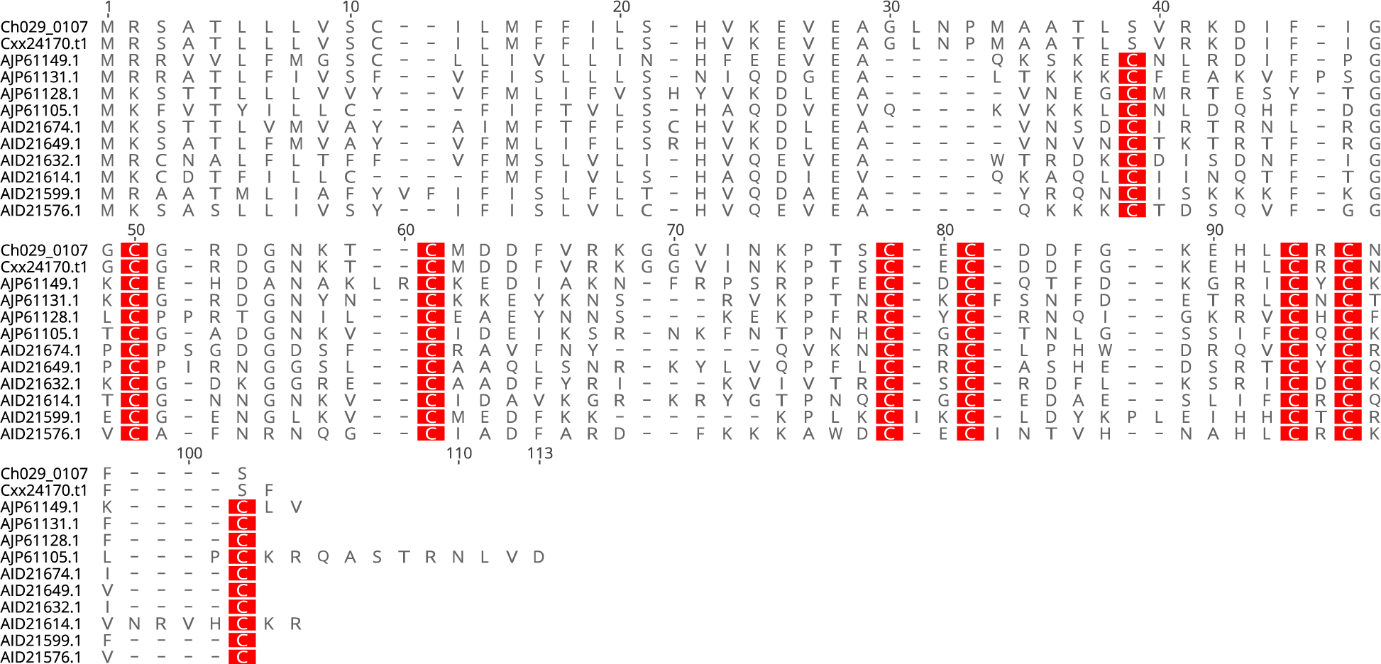


**Supplementary Figure 10. Protein sequence alignment of S-locus SCR genes from *C. lasiocarpa*, *C. himalaica* and other close relatives.** Cysteine residues are highlighted in red, showing eight conserved sites important for structural and functional integrity of the protein.


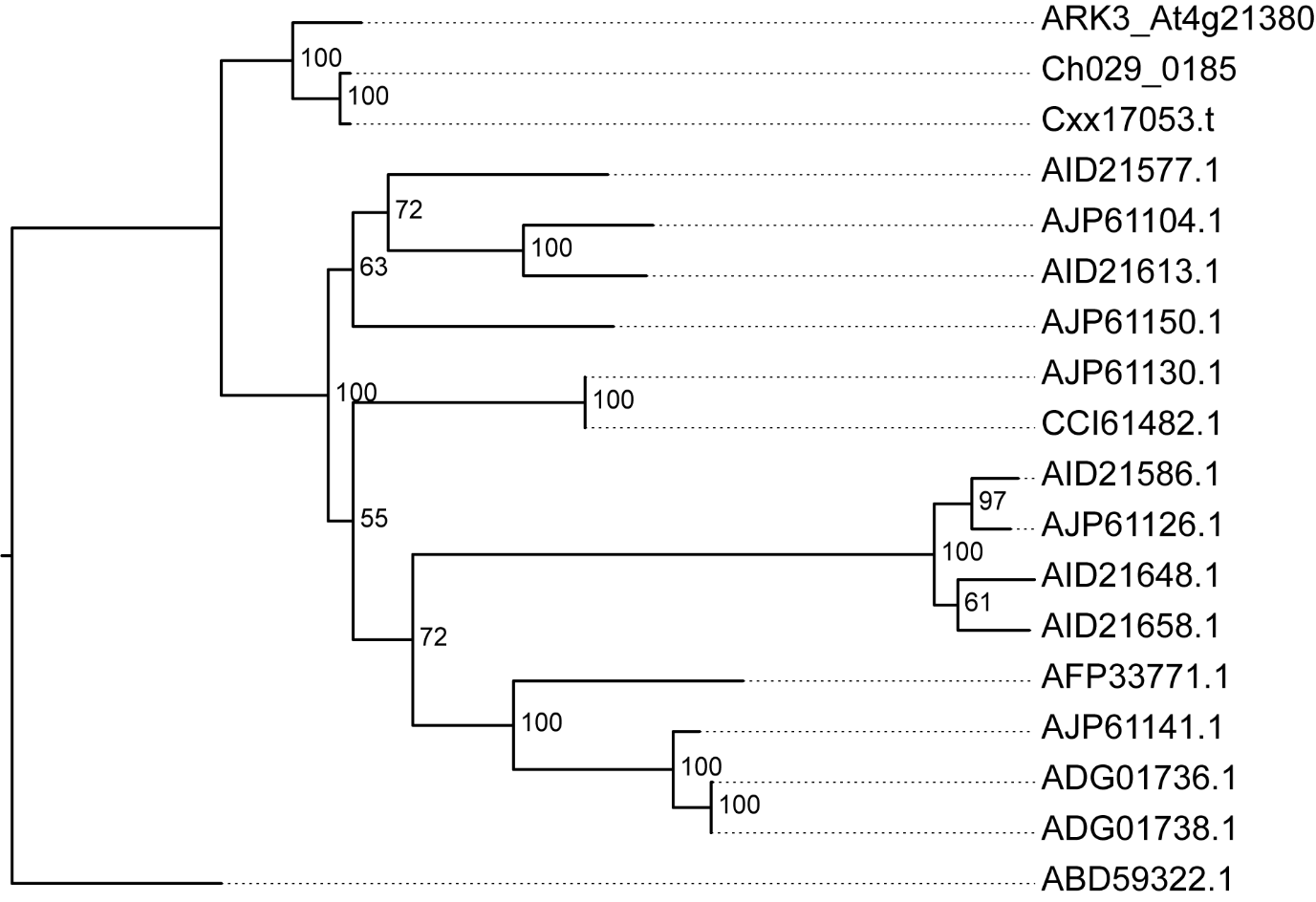


**Supplementary Figure 11. Maximum-likelihood phylogenetic tree of candidate ARK3 protein and ARK3 protein in *A. thaliana* and SRK proteins form *Arabidopsis*.**

**
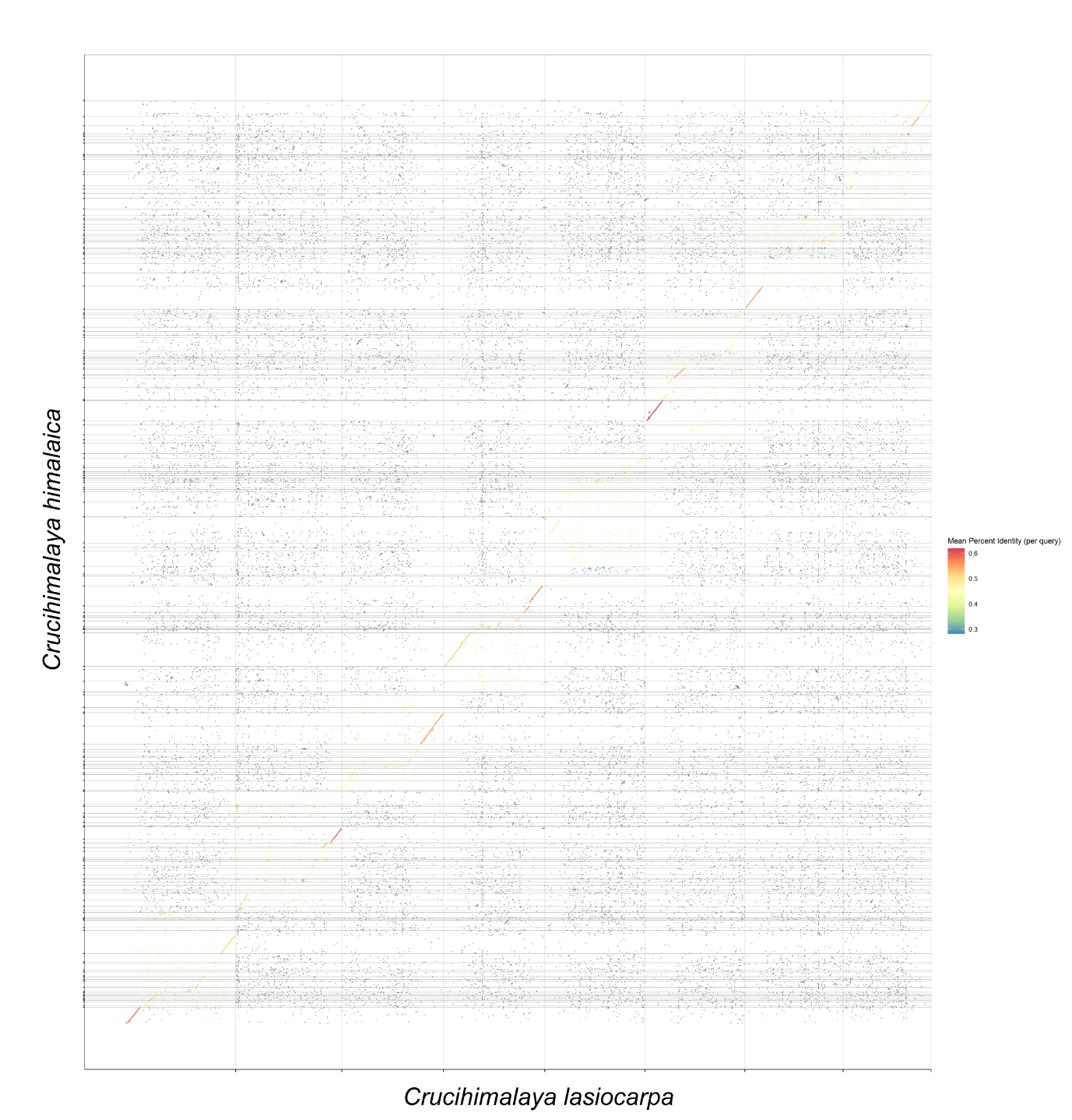
**

**Supplementary Figure 12. Collinearity dot plot of *C. lasiocarpa* and *C. himalaica* draw by minimap.**


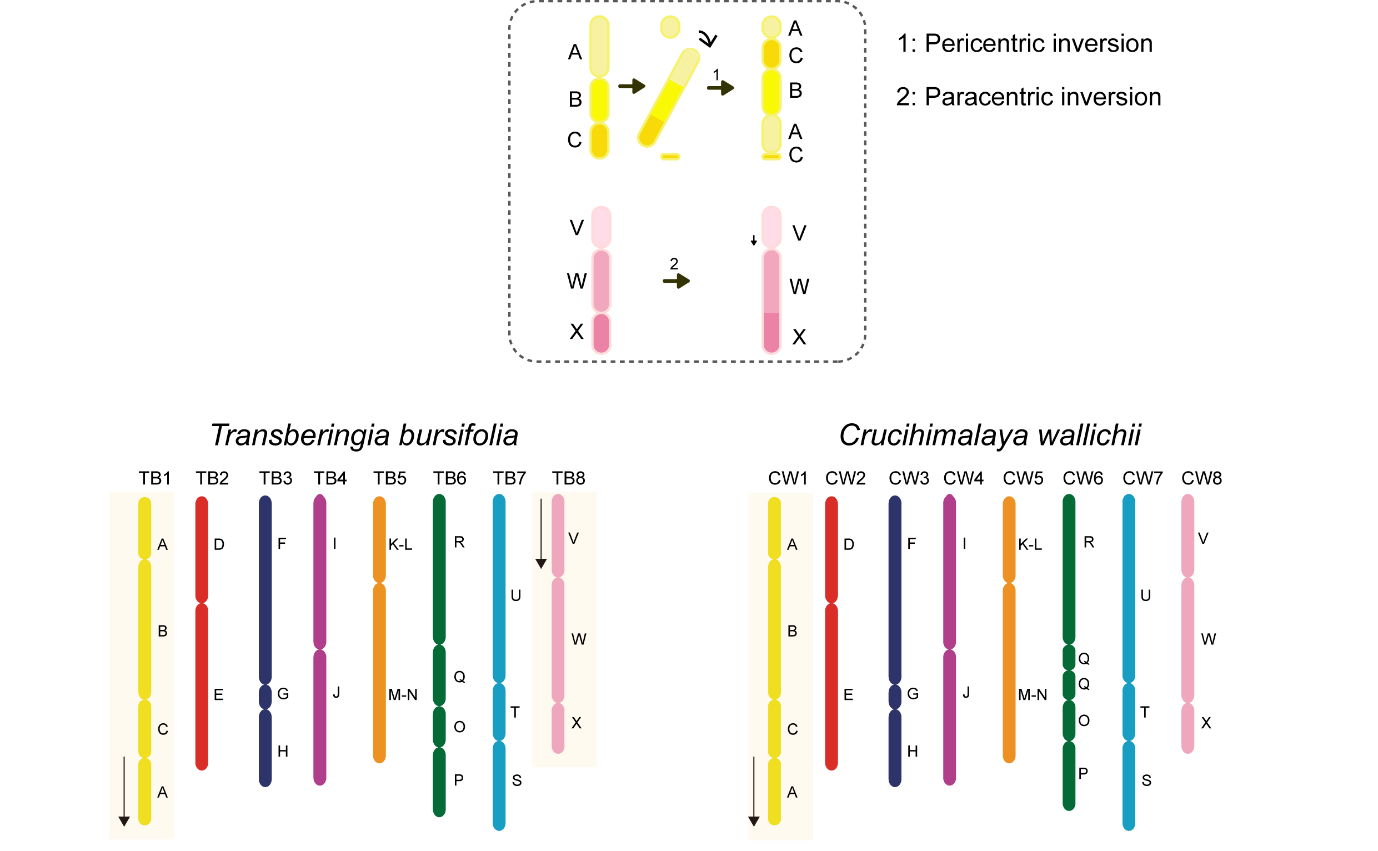


**Supplementary Figure 13. Karyotypes of *Transberingia bursifolia* (n = 8) and *Crucihimalaya wallichii* (n = 8)**.

**Supplementary Tables**

**Supplementary Table 1. Gene data sets used for gene prediction analysis.**

| **Species** | **Version** | **Data source/ GenBank accession number** |
| --- | --- | --- |
| *Aethionema arabicum* | 1.0 | NCBI: PRJNA202984 |
| *Arabidopsis lyrata* | 1.0 | https://phytozome.jgi.doe.gov |
| *Arabidopsis thaliana* | Tair10 | https://phytozome.jgi.doe.gov |
| *Boechera stricta* | 1.2 | https://phytozome.jgi.doe.gov |
| *Brassica rapa* | 3.0 | https://phytozome.jgi.doe.gov |
| *Capsella grandiflora* | 1.1 | https://phytozome.jgi.doe.gov |
| *Capsella rubella* | 1.1 | https://phytozome.jgi.doe.gov |
| *Carica papaya* | 0.4 | https://phytozome.jgi.doe.gov |
| *Leavenworthia alabamica* | 1.0 | NCBI: PRJNA202983 |
| *Tarenaya hassleriana* | 1.0 | NCBI: PRJNA202979 |

**Supplementary Table 2. Gene data sets used for comparative genomic analysis.**

| **Species** | **Version** | **Data source/ GenBank accession number** |
| --- | --- | --- |
| *Aethionema arabicum* | 1.0 | NCBI: PRJNA202984 |
| *Arabidopsis thaliana* | Tair10 | https://phytozome.jgi.doe.gov |
| *Capsella rubella* | 1.1 | https://phytozome.jgi.doe.gov |
| *Crucihimalaya himalaica* | 1.0 | NCBI: PRJNA521295 |
| *Crucihimalaya lasiocarpa* | 1.0 | NCBI: PRJNA763756 |
| *Eutrema heterophyllum* | 1.0 | NCBI: PKMM00000000 |
| *Eutrema salsugineum* | 1.0 | https://phytozome.jgi.doe.gov |
| *Eutrema yunnanense* | 1.0 | NCBI: PKML00000000 |

**Supplementary Table 3: Summary of DNA sequencing data.**

| **Library type** | **Platform** | | **Total Data (Gb)** | **Total Reads Number** | **Depth (X)** |
| --- | --- | --- | --- | --- | --- |
| Nanopore | | PromethION | 37.72 | 1,697,677 | 139 |
| Hi-C | | Illumina NovaSeq  Illumina NovaSeq | 54.99 | 362,595,252 | 104 |
| Short reads | |  | 18.00 | 122,398,250 | 53 |

**Supplementary Table 4: Summary of RNA sequencing data.**

| **Library type** | **Platform** | | **Tissue** | **Total Reads Number** | **Total Data (Gb)** |
| --- | --- | --- | --- | --- | --- |
| RNA-Seq | | MGI-2000 | Mature leaf | 48,040,804 | 5.7 |
| RNA-Seq | | MGI-2000 | Entire root | 46,936,876 | 5.7 |

**Supplementary Table 5: Summary of the functional annotation of predicted genes in *C. lasiocarpa*.**

| **Annotation database** | **Annotated number** | **Percentage (%)** |
| --- | --- | --- |
| Swiss-prot | 18,296 | 75.70 |
| Nr | 23,547 | 97.42 |
| InterPro | 22,745 | 94.10 |
| GO | 20,825 | 86.16 |
| KEGG Pathway | 8,630 | 35.70 |
| Total | 24,169 | 100.00 |

**Supplementary Table 6: Benchmarking Universal Single Copy Orthologs (BUSCO) analysis about *C. lasiocarpa* and *C. himalaica*.**

| Species | BUSCO annotation assessment |
| --- | --- |
| ***C. lasiocarpa*** | C:97.2% [D:1.2%], F:0.7%, M:2.1% n:1614 |
| *C. himalaica* | C:96% [D:16%], F:1.4%, M:2.1%, n:956 |

**Supplementary Table 7: Classification of repetitive elements in *C. larsiocarpa* genome.**

| Repeat type | number of elements | Repeat size (bp) | Percentage of genome (%) |
| --- | --- | --- | --- |
| SINEs | 634 | 166,849 | 0.07% |
| LINEs | 5,314 | 5,393,478 | 2.11% |
| LTR elements | 40,246 | 69,103,832 | 27.01% |
| DNA elements | 37,521 | 27,649,990 | 10.81% |
| Unclassified | 40,356 | 28,737,343 | 11.23% |
| total |  |  | 51.23% |

**Supplementary Table 8: KEGG functional annotation of the significantly expanded gene families in *C. lasiocarpa* and *C. himalaica*.**

| Gene families | KEGG terms | Input number | Background number | P-Value |
| --- | --- | --- | --- | --- |
| Expanded gene families | Glutathione metabolism | 2 | 147 | 1.47E-02 |
|  | Protein processing in endoplasmic reticulum | 2 | 326 | 6.20E-02 |
|  | Fatty acid biosynthesis | 1 | 55 | 6.69E-02 |
|  | Nucleotide excision repair | 1 | 101 | 1.19E-01 |
|  | RNA degradation | 1 | 160 | 1.81E-01 |
|  | mRNA surveillance pathway | 1 | 173 | 1.94E-01 |
|  | Amino sugar and nucleotide sugar metabolism | 1 | 219 | 2.39E-01 |
|  | RNA transport | 1 | 270 | 2.85E-01 |

**Supplementary Table 9: KEGG functional annotation of the significantly contracted gene families in *C. lasiocarpa* and *C. himalaica*.**

| Gene families | Functional terms | Input number | Background number | P-Value |
| --- | --- | --- | --- | --- |
| Contracted gene families | Camalexin biosynthesis | 4 | 39 | 1.29E-06 |
|  | Triacylglycerol degradation | 3 | 100 | 9.59E-04 |
|  | Flavone and flavonol biosynthesis | 1 | 9 | 1.85E-02 |
|  | Esterified suberin biosynthesis | 1 | 10 | 2.03E-02 |
|  | Farnesene biosynthesis | 1 | 16 | 3.12E-02 |
|  | Brassinosteroids inactivation | 1 | 19 | 3.66E-02 |
|  | Other glycan degradation | 1 | 23 | 4.38E-02 |
|  | Indole-3-acetate biosynthesis II | 1 | 25 | 4.73E-02 |

**Supplementary Table 10: PSGs with function description in Swissprot database.**

| **Gene ID** | **Swiss-Prot Annotation** | **Gene function description** |
| --- | --- | --- |
| Cxx00035.t1 | ATI1_ARATH | Involved in a special stress-induced plastid-to-vacuole protein trafficking pathway. Interacts with ATG8F in plastid bodies to subsequently enable their delivery to the vacuole by an autophagic pathway. Interacts with the plastid proteins APE1 and PSBS/NPQ4 and may recruit them as cargo into plastid bodies that may be recognized by the autophagy machinery for degradation in the vacuole. Involved in the alleviation of damage caused by salt stress during plant development, probably through its involvement in plastid-to-vacuole and ER-to-vacuole trafficking. Plays a role in seed germination in response to exogenous abscisic acid (ABA) treatment. |
| Cxx00230.t1 | PER28_ARATH | Removal of H(2)O(2), oxidation of toxic reductants, biosynthesis and degradation of lignin, suberization, auxin catabolism, response to environmental stresses such as wounding, pathogen attack and oxidative stress. These functions might be dependent on each isozyme/isoform in each plant tissue. |
| Cxx00238.t1 | PFI_PTIFI | Involved in the biosynthesis of conjugated triene- containing fatty acids. Catalyzes the isomerization of a wide range of substrates containing three or more methylene interrupted olefins into a Z,E,E conjugated triene functionality. May be involved in a stress tolerance mechanism as response to intertidal habitats with direct sunlight, desiccation and high temperature. In vitro substrates include arachidonic acid ((5Z,8Z,11Z,14Z)- eicosatetraenoic acid), EPA ((5Z,8Z, 11Z,14Z,17Z)-eicosapentaenoic acid), DHA ((4Z,7Z,10Z,13Z,16Z,19Z)-docosahexenoic acid), adrenic acid ((7Z,10Z,13Z,16Z)-docosatetraenoic acid), anandamide (arachidonyl-N-ethanolamide) and eicosatrienoic acid ((5Z,8Z,11Z)- eicosatrienoic acid). Gamma-linolenic acid (18:3 6Z,9Z,12Z) and dihomo-gamma-linolenic acid (20:3 8Z,11Z,14Z) are transformed into mixtures of conjugated diene and triene fatty acids, linoleic acid is only transformed to a conjugated diene. |
| Cxx00387.t1 | BETV6_BETPN | Oxidoreductase involved in lignan biosynthesis. Catalyzes the NADPH-dependent reduction of phenylcoumaran benzylic ethers. Converts dehydrodiconiferyl alcohol (DDC) to isodihydrodehydrodiconiferyl alcohol (IDDDC). |
| Cxx00446.t1 | PHL2_ARATH | Transcriptional activator. Acts redundantly with PHR1 as a key component of the central regulatory system controlling transcriptional responses to Pi starvation. Binds in a sequence-specific manner to phosphate starvation-regulated promoters. |
| Cxx00501.t1 | NAC55_ARATH | Transcription factors that bind specifically to the 5'- CATGTG-3' motif. |
| Cxx00666.t1 | IPT9_ARATH | Catalyzes the transfer of a dimethylallyl group onto the adenine at position 37 in tRNAs that read codons beginning with uridine, leading to the formation of N6-(dimethylallyl)adenosine (i(6)A). Involved in the cis-type cytokinin biosynthesis. |
| Cxx00721.t1 | NO70_SOYBN | Possible sulfate transporter. |
| Cxx00839.t1 | HS17A_ARATH | Possesses chaperone activity. |
| Cxx00897.t1 | PIRL2_ARATH | Leucine-rich repeat protein that likely mediates protein interactions, possibly in the context of signal transduction. |
| Cxx00935.t1 | RER5_ARATH | May play a role in leaf development. |
| Cxx01061.t1 | AARE1_ORYSJ | Catalyzes the hydrolysis of the N-terminal peptide bond of an N-acetylated peptide to generate an N-acetylated amino acid and a peptide with a free N-terminus. |
| Cxx01219.t1 | GAT19_ARATH | Transcriptional regulator that specifically binds 5'- GATA-3' or 5'-GAT-3' motifs within gene promoters. Regulates both flower and shoot apical meristem (SAM) development, especially for establishing organ boundaries in shoots and flowers, probably by controlling the number and position of WUS-expressing cells (PubMed:23335616, PubMed:25077795). |
| Cxx01264.t3 | CLCC_ARATH | Voltage-gated chloride channel. |
| Cxx01311.t1 | GAT29_ARATH | Transcriptional regulator that specifically binds 5'- GATA-3' or 5'-GAT-3' motifs within gene promoters. |
| Cxx01503.t1 | LEA50_ARATH | LEA proteins are late embryonic proteins abundant in higher plant seed embryos. The function of those proteins is not known. |
| Cxx01760.t1 | VA722_ARATH | Involved in the targeting and/or fusion of transport vesicles to their target membrane. |
| Cxx01906.t1 | EBSL_ARATH | Chromatin remodeling factor that binds to methylated histone (e.g. H3K4me2/3) to prevent their acetylation (e.g. H3K9K14Ac), likely by recruiting histone deacetylase (HDAC) complexes, and thus regulate the transcription of target genes. |
| Cxx02021.t1 | APD3_ARATH | Involved in pollen mitosis II (PMII) regulation during male gametogenesis. |
| Cxx02040.t1 | CARDE_CYNCA | Aspartic protease with a high preference for bonds between hydrophobic residues. |
| Cxx02188.t1 | CSCLE_ARATH | Acts as an osmosensitive calcium-permeable cation channel. |
| Cxx02237.t2 | MLH3_ARATH | Involved in DNA mismatch repair (MMR), correcting insertion-deletion loops (IDLs) resulting from DNA replication, DNA damage or from recombination events between non-identical sequences during meiosis. Component of the MutLbeta heterodimer, which probably forms a ternary complex with the MutSbeta heterodimer that initially recognizes the DNA mismatches. This complex is thought to be responsible for directing the downsteam MMR events, including strand discrimination, excision, and resynthesis. Plays a major role in promoting meiotic crossing-over and is involved in maintaining the genetic stability of simple sequence repeats by correction of frameshift intermediates. |
| Cxx02288.t1 | SRGT1_CHLRE | Glycosyltransferase involved in the O-galactosylation of several proteins including extensins. Catalyzes the transfer of alpha-galactosyl to Ser residues. Hydroxylation of proline residues adjacent to the serine acceptor is required for activity. Utilizes selectively UDP-galactose as a donor nucleotide sugar. |
| Cxx02350.t1 | RER3_ARATH | May play a role in leaf development. Required for leaf mesophyll cell division in the early stages of leaf organogenesis. |
| Cxx02366.t1 | SRS8_ARATH | Transcription activator that binds DNA on 5'-ACTCTAC-3' and promotes auxin homeostasis-regulating gene expression (e.g. YUC genes), as well as genes affecting stamen development, cell expansion and timing of flowering. Synergistically with other SHI- related proteins, regulates gynoecium, stamen and leaf development in a dose-dependent manner, controlling apical-basal patterning. Promotes style and stigma formation, and influence vascular development during gynoecium development. May also have a role in the formation and/or maintenance of the shoot apical meristem (SAM). |
| Cxx02470.t1 | ALF4_ARATH | Required for the initiation of lateral roots independent from auxin signaling. May function in maintaining the pericycle in the mitotically competent state needed for lateral root formation. |
| Cxx02471.t1 | TR120_ORYSJ | Specific subunit of the TRAPP II complex, a highly conserved vesicle tethering complex that is required for the proper transport of proteins in post-Golgi trafficking pathways to the growing cell plate in mitotic active cells. |
| Cxx02556.t1 | MAD21_ORYSJ | Probable transcription factor. |
| Cxx02618.t1 | LPAAT_ARATH | Lysophosphatidic acid acyltransferase which functions in phosphatidic acid biosynthesis. Is highly specific for lysophosphatidic acid and able to use different acyl-CoA donors. May regulate neutral lipid accumulation and participate in the regulation of lipid turnover in vegetative cells. Possesses additional triacylglycerol lipase and phospholipase A2 activities in vitro. Is not active as esterase or lysophospholipase. |
| Cxx02785.t1 | APF1_ARATH | Aspartyl protease. Not able to cleave BAG6. |
| Cxx02905.t5 | ATE2_ARATH | Involved in the post-translational conjugation of arginine to the N-terminal aspartate or glutamate of a protein. This arginylation is required for degradation of the protein via the ubiquitin pathway. Component of the N-end rule pathway with ATE1 and PRT6 (PubMed:19255443, PubMed:19620738, PubMed:22020282). The N-end rule pathway regulates seed after-ripening, seedling sugar sensitivity, seedling lipid breakdown, and abscisic acid (ABA) sensitivity of germination. The end-rule pathway regulates various aspects of leaf and shoot development. Involved in the oxygen-dependent N-arginylation of RAP2-12, an activator of hypoxic gene expression. This N- terminal modification leads to ubiquitination by PRT6 and subsequent degradation of RAP2-12 under aerobic conditions. Involved in disease resistance. The end-rule pathway plays a role in regulating the timing and amplitude of the immune response following infection with the bacterial pathogen Pseudomonas syringae pv tomato. Regulates the biosynthesis of plant- defense metabolites such as glucosinolates, and the biosynthesis and response to the phytohormone jasmonate (JA), which plays a key role in plant immunity. |
| Cxx02958.t1 | ZDH17_ARATH | Palmitoyl acyltransferase. |
| Cxx03075.t1 | SD17_ARATH | Involved in the regulation of cellular expansion and differentiation. Mediates subcellular relocalization of PUB9 from nucleus to plasma membrane in a protein-phosphorylation-dependent manner. May be involved in the abscisic acid-mediated signaling pathway, at least during germination. |
| Cxx03172.t1 | TRN2_HYONI | Catalyzes the stereospecific reduction of tropinone to pseudotropine. |
| Cxx03290.t1 | ATL31_ORYSJ | Possesses E3 ubiquitin-protein ligase in vitro. |
| Cxx03524.t1 | ASHH4_ARATH | Histone methyltransferase. |
| Cxx03550.t1 | ARR12_ARATH | Transcriptional activator that binds specifically to the DNA sequence 5'-[AG]GATT-3'. Functions as a response regulator involved in His-to-Asp phosphorelay signal transduction system. Phosphorylation of the Asp residue in the receiver domain activates the ability of the protein to promote the transcription of target genes. Could directly activate some type-A response regulators in response to cytokinins. Involved in the root- meristem size determination through the regulation of cell differentiation. Involved in activating SHY2 during meristem growth and controls PIN expression via activation of SHY2. |
| Cxx03911.t1 | IDD7_ARATH | Probable transcription factor. |
| Cxx04418.t1 | RVE4_ARATH | Probable transcription factor. |
| Cxx04426.t1 | PRR37_ORYSI | Controls photoperiodic flowering response. Seems to be one of the component of the circadian clock. Expression of several members of the ARR-like family is controlled by circadian rhythm. The particular coordinated sequential expression of PRR73, PRR37, PRR95, PRR59 and PPR1 result to circadian waves that may be at the basis of the endogenous circadian clock. |
| Cxx04625.t1 | BTR1_ARATH | Negative regulator of tomato mosaic virus (ToMV) multiplication, but has no effect on the multiplication of cucumber mosaic virus (CMV). Limits the spreading of the virus. Isoform BTR1S: binds preferentially and directly to the 5'terminal region of ToMV genomic RNA, and affects the efficiency of translation rather than mRNA stability. |
| Cxx04713.t1 | RBL11_ARATH | Rhomboid-type serine protease that catalyzes intramembrane proteolysis. May be involved in TIC22 processing during its import. |
| Cxx04887.t2 | DEK1_MAIZE | Essential protease involved in epiderm development. Required for aleurone cell development in the endosperm probably by maintaining and restricting the aleurone and embryonic epidermal L1 cell-layer fates as well as meristems organization. Involved in the maintenance of adaxial/abaxial axis information in developing leaves, probably by regulating cell proliferation and expansion. Does not need calcium ions to be active. |
| Cxx05117.t1 | PSDE_ARATH | Metalloprotease component of the 26S proteasome that specifically cleaves 'Lys-63'-linked polyubiquitin chains. The 26S proteasome is involved in the ATP-dependent degradation of ubiquitinated proteins. The function of the 'Lys-63'-specific deubiquitination of the proteasome is unclear. |
| Cxx05183.t1 | YSL7_ARATH | May be involved in the transport of nicotianamine- chelated metals. |
| Cxx05242.t1 | G6PD_SOLTU | Catalyzes the rate-limiting step of the oxidative pentose-phosphate pathway, which represents a route for the dissimilation of carbohydrates besides glycolysis. The main function of this enzyme is to generate NADPH for reductive biosyntheses. |
| Cxx05455.t1 | CHX6A_ARATH | May operate as a cation/H(+) antiporter. |
| Cxx05519.t1 | TOL5_ARATH | Might contribute to the loading of the ESCRT machinery. |
| Cxx05707.t1 | ERS1_ORYSI | Ethylene receptor related to bacterial two-component regulators. Acts as a redundant negative regulator of ethylene signaling. |
| Cxx05783.t1 | AHP6_ARATH | Functions as two-component phosphorelay mediators between cytokinin sensor histidine kinases and response regulators (B-type ARRs). Plays an important role in propagating cytokinin signal transduction. |
| Cxx05814.t2 | D27_ORYSJ | Involved in strigolactones biosynthesis by catalyzing the isomerization of the C9-C10 double bond in all-trans-beta- carotene leading to 9-cis-beta-carotene and providing the substrate for CCD7. Strigolactones are hormones that inhibit tillering and shoot branching through the MAX-dependent pathway, contribute to the regulation of shoot architectural response to phosphate-limiting conditions and function as rhizosphere signal that stimulates hyphal branching of arbuscular mycorrhizal fungi and trigger seed germination of root parasitic weeds. |
| Cxx05861.t1 | ASHH3_ARATH | Histone methyltransferase. |
| Cxx05982.t1 | CHS7_SORBI | The primary product of this enzyme is 4,2',4',6'- tetrahydroxychalcone (also termed naringenin-chalcone or chalcone) which can under specific conditions spontaneously isomerize into naringenin. |
| Cxx05988.t1 | LCMT1_ARATH | Methylates the carboxyl group of the C-terminal leucine residue of protein phosphatase 2A (PP2A) catalytic subunits to form alpha-leucine ester residues (Probable). Involved in brassinosteroid (BR) signaling. Plays a negative role in BR signaling pathway. Functions as a positive regulator of BRI1 receptor-kinase degradation. Methylates PP2A, thus facilitating its association with activated BRI1. This leads to receptor dephosphorylation and degradation, and thus to the termination of BR signaling. May act upstream of ASK7/BIN2. Involved in methylation of PP2A during environemental stress responses. |
| Cxx06226.t1 | TPPC_ARATH | Removes the phosphate from trehalose 6-phosphate to produce free trehalose. Trehalose accumulation in plant may improve abiotic stress tolerance |
| Cxx06295.t1 | PIN1_DIGLA | Prolyl cis/trans isomerase with specificity for phospho- Ser-Pro bonds. |
| Cxx06379.t1 | EXOL6_ARATH | May play a role in a brassinosteroid-dependent regulation of growth and development. |
| Cxx06645.t1 | CHX12_ARATH | May operate as a cation/H(+) antiporter. |
| Cxx06672.t2 | PIP_ARATH | Specifically catalyzes the removal of N-terminal proline residues from peptides. |
| Cxx06719.t1 | SKIP1_ARATH | Component of SCF(ASK-cullin-F-box) E3 ubiquitin ligase complexes, which may mediate the ubiquitination and subsequent proteasomal degradation of target proteins. |
| Cxx07155.t1 | FLZ17_ARATH | May act as an adapter to facilitate the interaction of SnRK1 complex with effector proteins, conferring tissue- and stimulus-type specific differences in the SnRK1 regulation pathway. |
| Cxx07331.t1 | MSH1_ARATH | DNA mismatch repair protein specifically involved in maintenance of mitochondrial genome configuration by controlling specific rearranged portion. Functions by suppressing asymmetric recombination at some repeat pairs. |
| Cxx07449.t1 | ASC1_SOLLC | Mediates resistance to sphinganine-analog mycotoxins (SAMs) by restoring the sphingolipid biosynthesis. Could salvage the transport of GPI-anchored proteins from the endoplasmic reticulum to the Golgi apparatus in ceramides-depleted cells after SAM exposure. |
| Cxx07519.t1 | TCP5_ARATH | Plays a pivotal role in the control of morphogenesis of shoot organs by negatively regulating the expression of boundary- specific genes such as CUC genes, probably through the induction of miRNA (e.g. miR164). Participates in ovule develpment. |
| Cxx07584.t1 | TI10A_ARATH | Repressor of jasmonate responses. Jasmonoyl-isoleucine (JA-Ile) specifically promotes COI1-TIFY10A/JAZ1 interaction. Interacts with COI1 and inositol pentakisphosphate to form a high- affinity jasmonates coreceptor. |
| Cxx07596.t1 | NAC2_ORYSJ | Transcription factor that possesses transactivation activity (PubMed:16924117, PubMed:20632034). Transcription activator involved in response to abiotic stresses. Plays a positive role during dehydration and salt stress. Binds specifically to the 5'-CATGTG-3' motif found in promoters of stress-responsive genes. |
| Cxx07653.t1 | PME23_ARATH | Acts in the modification of cell walls via demethylesterification of cell wall pectin. |
| Cxx07655.t1 | NAC2_CHLRE | Involved, directly or indirectly, in the processing of the chloroplast encoded psbD mRNA to its mature form, acting via the 5'-UTR of the psbD mRNA. The last 588 amino acids of the protein are sufficient to confer stability on the transcript in vivo. |
| Cxx07670.t1 | 4CL2_ARATH | Produces CoA thioesters of a variety of hydroxy- and methoxy-substituted cinnamic acids, which are used to synthesize several phenylpropanoid-derived compounds, including anthocyanins, flavonoids, isoflavonoids, coumarins, lignin, suberin and wall- bound phenolics. |
| Cxx07689.t1 | SWT12_ARATH | Mediates both low-affinity uptake and efflux of sugar across the plasma membrane. Involved in phloem loading by mediating export from parenchyma cells feeding H(+)-coupled import into the sieve element/companion cell complex, thus contributing to the sucrose migration from sites of synthesis in the mesophyll to the phloem (PubMed:22157085, PubMed:25988582). Contributes to seed filling by triggering sucrose efflux involved in the transfer of sugars from seed coat to embryos. |
| Cxx07754.t1 | EREL2_ARATH | Acts as an effector of RABF2A and RABF2B. Involved in vacuolar transport of storage proteins. Regulates membrane trafficking to protein storage vacuoles (PSVs) (Probable). Binds specifically to phosphatidylinositol 3- monophosphate (PtdIns3P). |
| Cxx07763.t1 | LURP1_ARATH | Involved in basal defense against virulent oomycetes. Might be related to the phospholipid scramblase and tubby-like superfamily of membrane tethered transcription factors. |
| Cxx07891.t1 | CCD1_ONCHC | Cleaves a variety of carotenoids symmetrically at both the 9-10 and 9'-10' double bonds. Catalyzes the formation of 4,9- dimethyldodeca-2,4,6,8,10-pentaene-1,12-dialdehyde and probably hydroxydihydro-beta-ionone from zeaxanthin. |
| Cxx07980.t1 | CAAT2_ARATH | Permease involved in the transport of the cationic amino acids. |
| Cxx08068.t1 | PHT1A_ORYSJ | Protein kinase that acts as a blue light photoreceptor in a signal-transduction pathway for phototropic responses. Regulates a wide range of physiological activities in plants that maximize the efficiency of photosynthesis, such as chloroplast relocations, stomata opening, and leaf expansion. |
| Cxx08093.t1 | ACT12_ARATH | Actins are highly conserved proteins that are involved in various types of cell motility and are ubiquitously expressed in all eukaryotic cells. Essential component of cell cytoskeleton; plays an important role in cytoplasmic streaming, cell shape determination, cell division, organelle movement and extension growth. This is considered as one of the reproductive actins. |
| Cxx08197.t1 | ELP4_ARATH | Acts as subunit of the RNA polymerase II elongator complex, which is a histone acetyltransferase component of the RNA polymerase II (Pol II) holoenzyme and is involved in transcriptional elongation. Promotes organs development by modulating cell division rate. May regulate mechanisms producing carbon assimilates or importing sucrose. Involved in the repression of the abscisic acid (ABA) signaling pathway during seed germination and seedling growth. Required for auxin distribution or signaling. Involved in oxidative stress signaling. Prevents anthocyanins accumulation. |
| Cxx08217.t1 | AZG2_ARATH | Transports natural purines (adenine and guanine) as well as purine analogs. Confers sensitivity to 8-azaadenine and 8- azaguanine (8-azg). |
| Cxx08227.t1 | PNAE_RAUSE | Catalyzes the hydrolysis of polyneuridine aldehyde into epi-vellosimine, which is the immediate precursor for the synthesis of the ajmaline. |
| Cxx08306.t1 | ASIL1_ARATH | Transcription repressor that binds specific DNA sequence such as the GT-box-like motif 5'-CGTGATT-3' in the AT2S3 promoter. Negative regulator of seed maturation genes during seed germination and seedling development. May target GT-box-containing embryonic genes by competing with the binding of transcriptional activators to this promoter region. Contributes to the maintenance and control of seed filling and may repress the maturation program during early embryogenesis. |
| Cxx08314.t1 | AGP30_ARATH | Proteoglycan required for the timing of seed germination. May function in the abscisic acid (ABA) response. |
| Cxx08315.t1 | FAB1A_ARATH | The PI(3,5)P2 regulatory complex regulates both the synthesis and turnover of phosphatidylinositol 3,5-bisphosphate (PtdIns(3,5)P2). Catalyzes the phosphorylation of phosphatidylinositol 3-phosphate on the fifth hydroxyl of the myo- inositol ring, to form phosphatidylinositol 3,5-bisphosphate. Plays an important role in maintenance of endomembrane homeostasis including endocytosis, vacuole formation, and vacuolar acidification processes. Required for development of viable pollen. Might mediate recycling of auxin transporters. |
| Cxx08355.t1 | PAO3_ORYSJ | Flavoenzyme involved in polyamine back-conversion. Catalyzes the oxidation of the secondary amino group of polyamines, such as spermine, spermidine and their acetyl derivatives. Substrate preference is spermidine > norspermine > thermospermine > N(1)-acetylspermine > spermine. No activity detected when putrescine is used as substrate. Plays an important role in the regulation of polyamine intracellular concentration (Probable). |
| Cxx08369.t1 | MYB54_ARATH | Transcription factor that regulates secondary cell wall (SCW) biosynthesis, especially in interfascicular and xylary fibers. |
| Cxx08426.t1 | PBL24_ARATH | May be involved in plant defense signaling. |
| Cxx08473.t1 | SWI3C_ARATH | Component of a multiprotein complex equivalent of the SWI/SNF complex, an ATP-dependent chromatin-remodeling complex, which is required for the positive and negative regulation of gene expression of a large number of genes. It changes chromatin structure by altering DNA-histone contacts within a nucleosome, leading eventually to a change in nucleosome position, thus facilitating or repressing binding of gene-specific transcription factors. |
| Cxx08534.t1 | COP1_ARATH | E3 ubiquitin-protein ligase that acts as a repressor of photomorphogenesis and as an activator of etiolation in darkness. E3 ubiquitin ligases accept ubiquitin from an E2 ubiquitin- conjugating enzyme in the form of a thioester and then directly transfers the ubiquitin to targeted substrates. Represses photomorphogenesis in darkness by mediating ubiquitination and subsequent proteasomal degradation of light-induced transcription factors such as HY5, HYH and LAF1. Down-regulates MYB21, probably via ubiquitination process. Light stimuli abrogate the repression of photomorphogenesis, possibly due to its localization to the cytoplasm. Could play a role in switching between skotomorphogenetic and photomorphogenetic pathways. Mediates the ubiquitination-dependent degradation of HY5 in the darkness during seedling development (e.g. hypocotyl growth). Represses CIP7 in darkness. |
| Cxx08555.t2 | ULP2B_ARATH | Protease that catalyzes two essential functions in the SUMO pathway: processing of full-length SUMOs to their mature forms and deconjugation of SUMO from targeted proteins. |
| Cxx08658.t1 | INVF_ARATH | Invertase that cleaves sucrose into glucose and fructose. |
| Cxx08660.t1 | HFR1_ARATH | Atypical bHLH transcription factor that regulates photomorphogenesis through modulation of phytochrome (e.g. PHYA) and cryptochrome signalings (Ref.4, PubMed:11090209, PubMed:10995393, PubMed:19482971). Suppresses the transcriptional regulation activity of PIF4 by forming non-DNA-binding heterodimer. |
| Cxx08667.t1 | ARIA_ARATH | May act as a substrate-specific adapter of an E3 ubiquitin-protein ligase complex (CUL3-RBX1-BTB) which mediates the ubiquitination and subsequent proteasomal degradation of target proteins. Acts as a positive regulator of ABA response via the modulation of the transcriptional activity of ABF2, a transcription factor which controls ABA-dependent gene expression via the G-box-type ABA-responsive elements. Negative regulator of seed germination and young seedling growth. |
| Cxx08670.t1 | NFYA1_ARATH | Stimulates the transcription of various genes by recognizing and binding to a CCAAT motif in promoters. |
| Cxx08771.t1 | NOL9_ARATH | Polynucleotide 5'-kinase involved in rRNA processing. |
| Cxx09079.t1 | RRFC_ORYSI | Responsible for the release of ribosomes from messenger RNA at the termination of chloroplastic protein biosynthesis. |
| Cxx09094.t1 | GRXS8_ARATH | May only reduce GSH-thiol disulfides, but not protein disulfides. |
| Cxx09134.t1 | CSI2_ARATH | Regulator of the microtubular cytoskeleton. Microtubule- associated protein involved in the association of cellulase synthase (CESA) complexes (CSCs) and cortical microtubules. Promotes dynamics of CSCs in the plasma membrane. Regulates primary cell wall biosynthesis and cellulose microfibrils organization. |
| Cxx09231.t1 | GT32_ORYSJ | Involved in the synthesis of glucuronoxylan hemicellulose in secondary cell walls. |
| Cxx09240.t1 | GBB_SOLTU | Guanine nucleotide-binding proteins (G proteins) are involved as a modulator or transducer in various transmembrane signaling systems. The beta and gamma chains are required for the GTPase activity, for replacement of GDP by GTP, and for G protein- effector interaction. |
| Cxx09344.t1 | PUB45_ARATH | Functions as an E3 ubiquitin ligase. |
| Cxx09694.t1 | RLK7_ARATH | Plays a role in pattern-triggered immunity (PTI) signaling induced by pathogen-associated molecular patterns (PAMPs). Acts as a receptor for PIP1 defense peptide. PIP1 is an endogenous secreted peptide that acts as elicitor of immune response and positive regulator of defense response. Involved in the control of seed germination speed, in tolerance to oxidative stress and in maintaining seed longevity. |
| Cxx09737.t1 | PRP19_ORYSJ | Probable ubiquitin-protein ligase which is mainly involved pre-mRNA splicing and DNA repair. |
| Cxx09759.t1 | EDA40_ARATH | Probable E3 ubiquitin-protein ligase involved in female gametophyte development. Required for fusion of polar nuclei in the embryo sac. |
| Cxx09778.t1 | FLA1_ARATH | May be a cell surface adhesion protein. |
| Cxx09999.t1 | E13B_MAIZE | Is thought to be an important plant defense-related product against fungal pathogens. |
| Cxx10004.t2 | FIPS3_ARATH | Component of the cleavage and polyadenylation specificity factor (CPSF) complex that plays a key role in pre- mRNA 3'-end formation, recognizing the AAUAAA signal sequence and interacting with poly(A) polymerase and other factors to bring about cleavage and poly(A) addition. FIP1L1 contributes to poly(A) site recognition and stimulates poly(A) addition. Binds to U-rich RNA sequence elements surrounding the poly(A) site. May act to tether poly(A) polymerase to the CPSF complex. |
| Cxx10027.t2 | GLO19_ORYSI | Aldehyde decarbonylase involved in the conversion of aldehydes to alkanes. Core component of a very-long-chain alkane synthesis complex. |
| Cxx10043.t1 | MYB63_ARATH | Transcriptional activator that binds DNA to the AC cis- elements 5'-ACCTACC-3', 5'-ACCAACC-3' and 5'-ACCTAAC-3' of promoters and specifically activates lignin biosynthetic genes during secondary wall formation mediated by SND1. |
| Cxx10128.t1 | DPHS1_ARATH | Probable dual specificity phosphatase that binds and dephosphorylates MPK18, modulating the organization and dynamics of cortical microtubules. Acts as negative regulator of abscisic acid (ABA) signaling during seed germination and light-induced stomata aperture. |
| Cxx10162.t2 | GBB_ARATH | Guanine nucleotide-binding proteins (G proteins) are involved as a modulator or transducer in various transmembrane signaling systems. The beta and gamma chains are required for the GTPase activity, for replacement of GDP by GTP, and for G protein- effector interaction. Together with GCR1 and GPA1, acts as a negative regulator of ABA during seed germination and early seedling development. The heterotrimeric G-protein controls defense responses to necrotrophic and vascular fungi probably by modulating cell wall-related genes expression (e.g. lower xylose content in cell walls); involved in resistance to fungal pathogens such as Alternaria brassicicola and Fusarium oxysporum. Modulates root architecture (e.g. lateral root formation). Acts with XGL3 in the positive regulation of root waving and root skewing. Involved in the asymmetric division of zygote and specification of apical and basal cell lineages. |
| Cxx10343.t1 | U91D1_STERE | May glycosylate diterpenes or flavonols in leaves. |
| Cxx10714.t1 | POR_AVESA | Phototransformation of protochlorophyllide (Pchlide) to chlorophyllide (Chlide). |
| Cxx11002.t1 | FRO8_ARATH | Ferric chelate reductase probably involved in iron reduction in leaf veins for transport. May participate in the transport of electrons to a Fe(3+) ion via FAD and heme intermediates. |
| Cxx11477.t1 | E13B_HEVBR | Possesses beta-1,3-endoglucanase activity in vitro. Is thought to be an important plant defense- related product against fungal pathogens (Probable). |
| Cxx11798.t1 | CLE43_ARATH | Extracellular signal peptide that regulates cell fate. |
| Cxx11815.t1 | ACFR3_ARATH | Two-heme-containing cytochrome. May catalyze ascorbate- dependent trans-membrane ferric-chelate reduction. |
| Cxx11873.t1 | OE64M_ARATH | Chaperone receptor mediating Hsp90-dependent protein targeting to mitochondria. |
| Cxx12206.t1 | ATL59_ARATH | E3 ubiquitin-protein ligase able to catalyze polyubiquitination with ubiquitin-conjugating enzyme E2 UBC8, UBC10, UBC11, and UBC34 in vitro. |
| Cxx12357.t1 | CRP1_MAIZE | Required for the translation of the chloroplast petA and petD mRNAs. Required for the processing of the petD mRNA from a polycistronic precursor. Binds with high affinity to the 5'-UTR of the chloroplastic petA transcript. Activates psaC and petA translation by binding their 5'-UTRs (PubMed:16141451, PubMed:23735295). |
| Cxx12459.t1 | CTF77_ARATH | One of the multiple factors required for polyadenylation and 3'-end cleavage of pre-mRNAs. Required for the targeted 3' processing of antisense transcripts that triggers transcriptional silencing of the corresponding sense gene. |
| Cxx12617.t1 | GOS12_ARATH | Involved in transport from the ER to the Golgi apparatus as well as in intra-Golgi transport. It belongs to a super-family of proteins called t-SNAREs or soluble NSF (N-ethylmaleimide- sensitive factor) attachment protein receptor. |
| Cxx12644.t1 | GAOX2_ARATH | Key oxidase enzyme in the biosynthesis of gibberellin that catalyzes the conversion of GA12 and GA53 to GA9 and GA20 respectively, via a three-step oxidation at C-20 of the GA skeleton. GA53 is less effectively oxidized than GA12, and GA25 is also formed as a minor product. Involved in the promotion of the floral transition, fertility and silique elongation, but plays only a minor role in elongation of seedling organs. Acts redundantly with GA20OX1. |
| Cxx12716.t1 | ACFR4_ARATH | Two-heme-containing cytochrome. May catalyze ascorbate- dependent trans-membrane ferric-chelate reduction. |
| Cxx12901.t1 | CYSKM_ARATH | Acts as a cysteine synthase. Plays a role in the sulfide detoxification in mitochondria. |
| Cxx13481.t1 | MYB4_ARATH | Transcription repressor involved in regulation of protection against UV. Mediates transcriptional repression of CYP73A5, the gene encoding trans-cinnamate 4-monooxygenase, thereby regulating the accumulation of the UV-protectant compound sinapoylmalate. |
| Cxx13550.t2 | NDHO_ARATH | NDH shuttles electrons from NAD(P)H:plastoquinone, via FMN and iron-sulfur (Fe-S) centers, to quinones in the photosynthetic chain and possibly in a chloroplast respiratory chain. The immediate electron acceptor for the enzyme in this species is believed to be plastoquinone. Couples the redox reaction to proton translocation, and thus conserves the redox energy in a proton gradient. |
| Cxx13636.t1 | RLK5_ARATH | Receptor with a dual specificity kinase activity acting on both serine/threonine- and tyrosine-containing substrates that controls floral organ abscission. May interact with the 'INFLORESCENCE DEFICIENT IN ABSCISSION' (IDA) ligands family. |
| Cxx13639.t1 | VP35C_ARATH | Plays a role in vesicular protein sorting. Component of the membrane-associated retromer complex which is essential in endosome-to-Golgi retrograde transport. Also involved in the efficient sorting of seed storage proteins. The VPS29-VPS26-VPS35 subcomplex may be involved in recycling of specific cargos from endosome to the plasma membrane. |
| Cxx13763.t1 | PBL25_ARATH | May be involved in plant defense signaling. |
| Cxx13831.t1 | TPPF_ARATH | Removes the phosphate from trehalose 6-phosphate to produce free trehalose. Trehalose accumulation in plant may improve abiotic stress tolerance. |
| Cxx14170.t2 | REMO_ARATH | Exhibits a non sequence-specific DNA-binding activity. |
| Cxx14378.t1 | JHS1_ARATH | Essential protein required during embryogenesis. Key enzyme involved in DNA replication and damage repair, shoot apical meristem (SAM) maintenance, and development. Involved in Okazaki fragments processing. Possesses different enzymatic activities, such as single-stranded DNA (ssDNA)-dependent ATPase, 5'-3' helicase and endonuclease activities. While the ATPase and endonuclease activities are well-defined and play a key role in Okazaki fragments processing and DSB repair, the 5'-3' DNA helicase activity is atypical: it cannot load onto its tracking strand internally and has an absolute free 5'-end requirement. |
| Cxx14458.t1 | OXA1L_ARATH | Probably required for the insertion of integral membrane proteins into the mitochondrial inner membrane. May participate in the activity and assembly of cytochrome oxidase. |
| Cxx14860.t1 | WRK19_ARATH | Transcription factor. Interacts specifically with the W box (5'-(T)TGAC[CT]-3'), a frequently occurring elicitor- responsive cis-acting element. May act also as a disease resistance protein with a serine/threonine-protein kinase activity. |
| Cxx14887.t1 | LHW_ARATH | Transcription activator that regulates root development; promotes the production of stele cells in roots. Coordinately controls the number of all vascular cell types by regulating the size of the pool of cells from which they arise. |
| Cxx14888.t1 | APBLC_ARATH | Subunit of clathrin-associated adaptor protein complex that plays a role in protein sorting in the late-Golgi/trans-Golgi network (TGN) and/or endosomes. The AP complexes mediate both the recruitment of clathrin to membranes and the recognition of sorting signals within the cytosolic tails of transmembrane cargo molecules. |
| Cxx14986.t2 | CIP7_ARATH | Exhibits transcriptional activation activity. Positive regulator of light-regulated genes, probably being a direct downstream target of COP1 for mediating light control of gene expression. |
| Cxx15073.t1 | RK176_ORYSJ | Functions downstream of CERK1 in the microbial peptidoglycans (PGNs) and fungal chitin signaling pathways that mediate innate immunity. Participates to the activation of defense genes during response to PGN and chitin. |
| Cxx15269.t1 | DLDH_PEA | Lipoamide dehydrogenase is a component of the glycine cleavage system as well as of the alpha-ketoacid dehydrogenase complexes. The pyruvate dehydrogenase complex contains multiple copies of three enzymatic components: pyruvate dehydrogenase (E1), dihydrolipoamide acetyltransferase (E2) and lipoamide dehydrogenase (E3). |
